# Ethnobotanical study of medicinal plants used against human ailments in Gubalafto District, Northern Ethiopia

**DOI:** 10.1186/s13002-017-0182-7

**Published:** 2017-10-04

**Authors:** Getnet Chekole

**Affiliations:** Department of Biology, College of Natural and Computational Sciences, University of Gondar, P.O. Box 196, Gondar, Ethiopia

**Keywords:** Healer, Indigenous knowledge, Traditional plant medicines

## Abstract

**Background:**

Traditional medicinal plant species documentation is very crucial in Ethiopia for biodiversity conservation, bioactive chemical extractions and indigenous knowledge retention. Having first observed the inhabitants of Gubalafto District (Northern Ethiopia), the author gathered, recorded, and documented the human traditional medicinal plant species and the associated indigenous knowledge.

**Methods:**

The study was conducted from February 2013 to January 2015 and used descriptive field survey design. Eighty-four informants were selected from seven study kebeles (sub-districts) in the District through purposive, snowball, and random sampling techniques. Both quantitative and qualitative data were collected through semi-structured interviews, guided field walks, demonstrations, and focus group discussions with the help of guided questions. Data were organized and analyzed by descriptive statistics with SPSS version 20 and Microsoft Office Excel 2007.

**Results:**

A total of 135 medicinal plant species within 120 genera and 64 families were documented. Among the species, *Ocimum lamiifolium* and *Rhamnus prinoides* scored the highest informant citations and fidelity level value, respectively. In the study area, Asteraceae with 8.1% and herbs with 50.4% plant species were the most used sources for their medicinal uses. A total of 65 ailments were identified as being treated by traditional medicinal plants, among which stomachache (abdominal health problems) was frequently reported. *Solanum incanum* was reported for the treatment of many of the reported diseases. The leaf, fresh parts, and crushed forms of the medicinal plants were the most preferred in remedy preparations. Oral application was the highest reported administration for 110 preparations. A majority of medicinal plant species existed in the wild without any particular conservation effort. Few informants (about 5%) had only brief notes about the traditional medicinal plants. Ninety percent of the respondents have learned indigenous medicinal plants knowledge from their family members and friends secretly. Orthodox Church schools were found the main place for 65% of healer’s indigenous knowledge origin and experiences. Elders, aged between 40 and 84 years, gave detailed descriptions about traditional medicinal plants.

**Conclusions:**

Traditional medicinal plants and associated indigenous knowledge are the main systems to maintain human health in Gubalafto District. But minimal conservation measures were recorded in the community. Thus, *in-situ* and *ex-situ* conservation practices and sustainable utilization are required in the District.

## Background

People have long histories on the uses of traditional medicinal plants for medical purposes in the world, and nowadays, this is highly actively promoted [1–3]. Evidence from Kibebew [4] showed that traditional medicines are used by 75–90% of the rural population in the world. The report from the World Health Organization [5] revealed that traditional medicinal plants were trusted primarily by 80% of the population in Africa. Traditional medicines are more liked in developing countries due to inadequate modern health services. In Ethiopia, the use of traditional plant medicines had been practiced since the ancient time [6]. In Northern Ethiopia, the major portions (87%) of the traditional medicines are coming from plant sources [7]. However, the traditional medicines are far from the expected level of uses, safety, and efficacy in the world [8, 9]. In Ethiopia, the bulk of the medicinal plants were collected from natural vegetation, and nowadays, natural vegetation is shrinking due to environment degradation and overuses. Therefore, it is necessary to document medicinal plant species for conservation and sustainable consumption. In addition, ethnobotanical studies on traditional medicinal plants are also the means to increase the capacity of the pharmaceutical industries. However, the documented medicinal plants are still limited when they are compared with the multi-cultural diversity of the people and the diverse flora in Ethiopia [10]. In Gubalafto District, the people live in places which are grouped into peaks, highlands, middle lands, and low lands. In such diverse environments, traditional medicinal plant species and their uses are expected to be more. However, no scientific documentation on the medicinal plant resources has so far been made in Gubalafto District. If any cultural changes take place in this community and the vegetation is degraded due to various factors, the knowledge of the people on the plant resource will vanish slowly. Moreover, some of the medicinal plant species may become extinct from the District before being documented and the people may lose their uses and their indigenous knowledge on them forever. Therefore, the ethnomedicinal study on the plants of Gubalafto is crucial in order to protect the plants under *ex-situ* and *in-situ* conservation and to preserve the associated indigenous knowledge in the District and beyond. Thus, the author documented the traditional medicinal plant species and the associated indigenous knowledge used for the treatment of human ailments in Gubalafto District.

## Methods

### Description of the study area

**Fig. 1:**
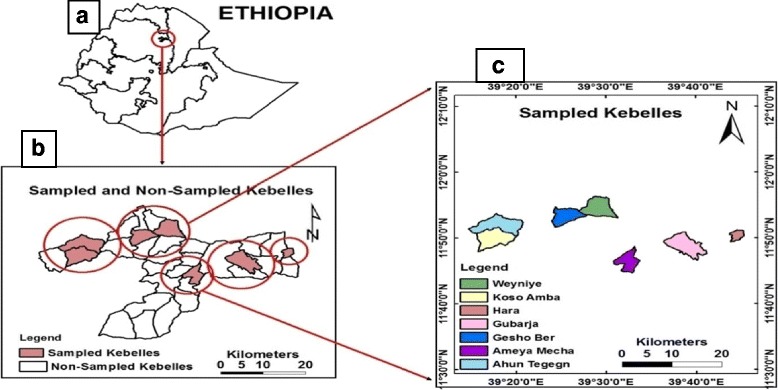
Map of the study area. **a** Location of Gubalafto District in Ethiopian. **b** Sampled and non-sampled kebeles in Gubalafto District. **c** List and site of sampled kebeles in Gubalafto District

Gubalafto District is found in North Wollo Zone, Amhara region, Ethiopia, by which Woldia is the main town of the District. Woldia is about 506 km far from Addis Ababa, and the main road cross it to Mekele, Dessie, Bahir Dar, and Lalibela towns. It consists of 34 kebeles, and it is situated between 39° 12′ 9″–39° 45′ 58″ East and 11° 34′ 54″–11° 58′ 59″ North (Fig. 1). The topography of the District ranges between 1100 and 3700 m above sea level (m.a.s.l) and mostly characterized by a chain of mountains (35%), undulations (30%), flats (20%), and gorges/valleys (15%). In addition, Gubalafto District is classified into four agro-ecological zones based on their altitudinal variation and climatic conditions. These are lowlands (1100–1500 m.a.s.l), middle lands (1500–2300 m.a.s.l), highlands (2300–3200), and peaks (3200–3700 m.a.s.l.). These agro-ecological zones were also known by the people as Kolla, Woina Dega, Dega, and Wurch, respectively. The mean values of annual temperature range from 7.5 to 22.8 °C and the rainfall from 22.8 to 203.7 mm in the form of bimodal (rain available in two seasons in a year) [11]. Gubalafto District has 139, 825 total population, of which 50.6% are men and 49.4% women [12]. About 3.49% of the population lives in the urban area, and the remaining 96.51%, in the rural area. People mainly depend on agriculture for their livelihoods. There are 51 health centers, of which 9 of them are private and 42 are governmental centers in the District. Acute febrile illness, acute upper respiratory infection, dyspepsia, diarrhea, pneumonia, helminthiasis, diseases of the muscular and skeletal system, trauma, urinary tract infection, infections of the skin, and subcutaneous tissues are the ten major morbidity diseases recorded in the District [13].

### Study sites and informant selection techniques

Study sites and informants were selected based on the information gathered from Gubalafto District administration office, health office, agricultural office, and other people in the study area via reconnaissance survey prior to the data collection. Accordingly, seven kebeles, namely Koso-amba, Ahuntegegn, Woyneye, Geshober, Amaye-mecha, Gubarja, and Hara were selected for data collection with purposive sampling method based on their agro-ecological conditions, the availability of traditional medicine practitioners, and vegetation covers (Fig. 1). Eighty-four informants (12 from each kebele) both males and females, whose ages ranged from 20 to 90 years were interviewed during the study. Informants were selected with purposive, snowball (non-probability), and random (probability) sampling techniques following previous publications [14–16]. The purposive sampling technique was used due to the fact that there were healers that had an official permission for their traditional healthcare practices. Information regarding healers was obtained from each sampled kebele health offices and other people. On the contrary, the snowball sampling technique was used to get hold of healers who had no official permission for their traditional medicinal practices and who were found through the suggestion of other interviewed informants confidentially. As the names of non-legalized healers were not registered in the governmental offices and the people hesitated to report their names freely, the use of a snowball sampling technique was useful.

In the same vein, additional traditional medicinal plant species and associated information were collected from general informants (non-healers) with random sampling techniques. General informants were ordinary people who were found in the study area for a long period of time and used their indigenous medicinal plant knowledge within their families. Hence, general informants were included as respondents to gather additional data and check the transfer of indigenous knowledge within the people. Generally, purposive and snowball samples were used to choose a total of 32 key informants, whereas 52 informants were selected using random sampling method.

It was necessary to follow some steps to contact informants. Firstly, legal supportive letters were obtained from Woldia University research and development office. The District administrators and health and agricultural officials in Gubalafto discussed with the researcher about the objectives of the study. Consequently, they permitted and gave a collaborative letter to conduct the study in the selected kebeles. Thirdly, they sent a letter of research permit for the kebele chairpersons and the personnel of health offices. Accordingly, the lists of officially recognized traditional healers of each kebele were given along with the diseases treated by the kebele health officers. Likewise, group discussions were performed with the people about the importance of the study. Finally, the kebele chairpersons and health officers conveyed messages for the people concerning their participation in the study. Field guides were used to contact healers and collect medicinal plant specimens.

### Data collection tools and procedures

Ethnobotanical data were collected from February 2013 to January 2015 in both rainy and dry seasons with a descriptive field survey design in which both qualitative and quantitative data were collected through semi-structured interviews, guided field walks, demonstrations, and focus group discussions. The semi-structure interviews were delivered with the help of pre-prepared questions in the English language. The items of the interview were done on the demographic characteristics of the informants included gender, age, job, educational level, religion, and category (either healers or general informants). Data were also focused on the uses of medicinal plants which incorporated local names, diseases treated, parts used, preparation methods, administration route/s, dosage, habits, and the habitats. The medicinal plant’s conservation practices, adverse effects (if any), taboos (if any), additional uses as wild food and livestock medicines (if any), and indigenous knowledge transfer systems were also included. Individual interviews were also conducted with each key informant for preference ranking exercise following methods used by previous researchers [14–16].

Data were also collected through demonstrations (plant interviews) in the cases of some females and aged male informants in their homes with the help of prior collected plant specimens collected from the field. This is because female and aged male informants had a minimal chance to move to far places to show medicinal plant specimens and their practices. Other informants also showed their medicinal plant practices in their homes and in the fields. One up to two focus group discussions were conducted in each sampled kebele with governmental officials, key informants, and other people with the help of general interview questions. Guided field walks were conducted with interviewed informants and other local indigenous people to search for additional medicinal plants in the wild and to collect medicinal plant voucher specimens. All interviewees were asked in the Amharic language, which is the language of the inhabitants of the study area, and the collected data were translated into English with the help of experts. Contact time and place were selected based on the interest of the informants.

The information about traditional medicinal plant specimens, consequently, was recorded in the notebook, and the plants were pressed with plant press and dried properly. The scientific names of the dried traditional medicinal plant specimen were identified in the National Herbarium (ETH) in Addis Ababa University, Ethiopia, by using published volumes of the Flora of Ethiopia and Eritrea [17–25], by the help of deposited authenticated specimens and taxonomists. Finally, the voucher specimens were deposited in ETH.

#### Data analysis

Data were organized and analyzed by Microsoft Office Excel spreadsheet 2007 and Statistical Package for Social Science (SPSS) version 20. Independent sample *t* test was computed to identify the number of medicinal plant species and associated uses reported by healers and general informants. Similarly, it was also used to identify the indigenous knowledge variation of males and females on the numbers of medicinal plant species and associated uses they mentioned. The ages of the informants were grouped into 20–39 (younger informants) and 40–84 (elder informants). Therefore, the variation in the numbers of medicinal plant species and associated uses reported within the two age groups were computed with independent sample *t* test.

Diseases recorded in this study were grouped into ten major categories with the help of physicians, and informant consensus factor (ICF) was calculated to determine the effectiveness of medicinal plants in each ailment category according to Heinrich et al. [26]. It was calculated by the formula: *ICF = Nur − Nt/Nur − 1*, where *Nur* refers to the number of use reports for a particular ailment category and *Nt* refers to the number of medicinal plant species used for a particular ailment category by all informants.

On the other hand, use value was also calculated to see the relative importance of each traditional medicinal plant species for treating diseases in the study area according to Phillips et al. [27]. It was calculated by the formula *UV* = *ΣUi*/*n* where *UV* stands for the total use value of the traditional medicinal plant species, whereas *U* refers to the number of use reports cited by each informant for a given plant species and *n* stands the total number of informants interviewed for a given plant species.

Fidelity level (FL) was computed to determine the FL values of the most frequently used plant species for treating a particular ailment according to Friedman et al. [28]. It was calculated by the formula *FL* = *Np*/*N* where *Np* stands for the number of use reports cited for a given species for a particular ailment and *N* refers to the total number of use reports cited for any given traditional medicinal plant species.

Furthermore, the preference ranking was determined by purposively drawn ten experienced key informants to prioritize the nine traditional medicinal plant species used for preventing bleeding according to Cotton [15]. Bleeding was preferred for ranking because it is a fatal and an emerging disease in the society.

## Results

### Respondents’ indigenous knowledge characteristics

Of the total 84 informants, the numbers of male participants were higher than those of females. Informants in the age range between 40 and 90 years were the highest in number (75%) and a little higher than half (53.6%) of the informants had gone through modern education (Table 1). The occupation of the informants showed that 83.3% of them were farmers, 6% were students, 3.6% were housewives, and some of the informants were represented with healers and jobless. From the total informants, 39.3% were key informants (healers) and 60.7% were general informants.

**Table 1:** Demographic details of the informants

Sex	Age group (in years)		Educational status		
	20–39	40–90	Illiterate	Religious education	Modern education
Male	13 (61.9%)	55 (87.3%)	13 (61.9%)	17 (94.4%)	38 (84.4%)
Female	8 (38.1%)	8 (12.7%)	8 (8.1%)	1 (5.6%)	7 (15.6%)
Total	21 (25%)	63 (75%)	21 (25%)	18 (21.4%)	45 (53.6)

The great majority of respondents (90%) reported that most of their knowledge was received from their family members and friends secretly. The secret practices of traditional medicines came from their ancestors. However, if it is not practiced secretly, they think that the potential of the medicinal values of the plants will be diminished. Furthermore, five Muslim healers acquired their knowledge through the local graduation (MIRKAN) system from other elder traditional medicine experts. Nevertheless, elders implement this kind of graduation after having observed their activity in practice. The five Muslim healers believed that traditional knowledge shared without local graduation system could not be usable and unsuitable for treating ailments. Furthermore, 60% of the healers reported that they learned their medicinal plant knowledge from their friends in the Orthodox Church schools.

Significant differences (*P* < 0.05) were obtained by independent sample *t* test between healers and general informants on the number of medicinal plant species and associated uses (Tables 2 and 3). From the respondent’s report, it was found that some key informants were noticed with few effective remedies that treated one or two ailments like specialized doctors in modern medicines. The test did not show a significant difference (*P* > 0.05) between male and female informants on the number of medicinal plant species they listed and associated uses reported. However, males reported the highest number of medicinal plant species and associated uses (Tables 2 and 3). The test also confirmed that there was no significant difference on the number of medicinal plant species mentioned by the two age groups (20–39 and 40–84 years) of informants and the respective uses they explained (Tables 2 and 3). However, elders whose ages were between 40 and 84 years noticed detailed descriptions and practical preparation techniques.

**Table 2:** Statistical test of significance and independent *t* test on the number of medicinal plants mentioned by informant groups in Gubalafto District

Parameters	Informant group	*N*	No. of plant species reported	Mean	*t* value**	*P* value
Informant category	General informant	51	303	5.94	− 4.334	0.000*
	Healer	33	541	16.39		
Gender	Male	68	706	10.38	0.529	0.598
	Female	16	138	8.63		
Age	Younger(20–39 years)	21	176	8.38	− 0.739	0.462
	Elder (40–84 years)	63	668	10.60		

*Significant difference (*P* < 0.05), **t (0.05) (two tailed), df = 82, *N*= number of respondents

**Table 3:** Statistical test of significance and independent *t* test on the number of medicinal plant use mentioned by informants in Gubalafto District

Parameters	Informant group	*N*	No. of plant species uses reported	Mean	*t* value**	*P* value
Informant category	General informant	51	268	5.25	− 4.406	0.000*
	Healer	33	424	12.85		
Gender	Male	68	581	8.54	0.676	0.501
	Female	16	111	6.94		
Age	Younger(20–39 years)	21	162	7.71	− 0.323	0.747
	Elder (40–84 years)	63	530	8.21		

*Significant difference (*P* < 0.05), **t (0.05) (two tailed), df = 82, *N*= number of respondents

On the other hand, traditional healers and members of the society reported that traditional medicinal practices are not encouraged by the kebele governmental offices, which are considered as illegal activities. Some of the healers also reported experiencing derogatory descriptions and scoldings by calling them witchcrafts, KITEL BETASH, SIRMASH, DEBTERA and ASMATEGNA, as explained by the traditional healers, which highly reduced their interests in the traditional medicine practices freely in the society. On the contrary, some of traditional healers are not interested in having governmental legal recognition for their traditional medicine practices due to income taxes that the government levy upon them.

Moreover, 95% of the informants reported that they have not seen any documented material about the traditional medicinal plants of their area and the associated uses. They transferred the knowledge through word of mouth (orally), and through time, they lost part of indigenous knowledge due to the difficulty of memorization. Only 5% of the respondents told that their indigenous knowledge on medicinal plants has been preserved in brief notebooks.

### Medicinal plant species of the study area

This study documented 135 traditional medicinal plant species belonging to 120 genera and 64 families, which are used to treat 65 human ailments (Appendix: Table 10). The plant family Asteraceae contributed the highest number of medicinal plant species (11) followed by Fabaceae, Lamiaceae, and Solanaceae with nine plant species each (Table 4 and Appendix: Table 10).

**Table 4:** Taxonomic diversity of medicinal plant species and their proportions

Families	No. of genera in each family	No. species in each family and genera	% of total
Asteraceae	10	11	8.1
Fabaceae and Lamiaceae	8	9	6.6
Solanaceae	7	9	6.6
Euphorbiaceae	5	6	4.4
Cucurbitaceae	4	4	2.9
Rutaceae	3	4	2.9
Apiaceae, Acanthaceae, Ranunculaceae, Rubiaceae, and Vitaceae	3	3	2.2
Moraceae	1	3	2.2
Eight families	2	2	1.5
Two families	1	2	1.5
41 families	1	1	0.7

Among the total documented medicinal plant species, *Solanum incanum* was used to treat the highest number of diseases (Table 5 and Appendix: Table 10). Stomachache (general abdominal problems), wound, febrile illness, swelling, and malaria were the commonly reported diseases, and these were treated with 1.6, 0.13, 0.07, 0.06, and 0.05% medicinal plant species, respectively. People in the study area give first priority for some traditional medicinal plant species to treat ailments than modern drugs. *Withania somnifera*, *Tragia brevipes*, *Cucumis ficifolius* and *Zingiber officinale*, *Ziziphus spina-christi*, *Salvia merjamie* and *Salvia nilotica*, and *Plantago lanceolata* and *Ruellia patula*, are found to be the most important medicinal plant species than the locally available modern drugs to treat swellings, dactylitis, ﻿﻿stomachache, dandruff, bleeding, herpes zoster, and occurrence of baldness, respectively. Likewise, healers reported that *Thalictrum rhynchocarpum*, *Ruta chalepensis*, and *Allium sativum* were mixed commonly when they prepared remedy from other traditional medicinal plant species. Besides, among the documented human medicinal plant species, *Carissa spinarum*, *Polygala sphenoptera*, *Cirsium englerianum*, *Verbascum sinaiticum*, and *Achyranthes aspera* are also used for the treatment of livestock diseases in Gubalafto District. Likewise, *Haplocarpha rueppelii*, *Urtica simensis*, *Grewia kakothamnos*, *Carissa spinarum*, *Cordia africana*, *Ficus vasta*, and *Ziziphus spina-christi* are used as food for humans in the wild. The informants in Hara (hot and relatively lowland in the District) also reported that the smashed leaves of *Ziziphus spina-christi* have been used to prevent the human corpse from rapid deterioration and bad smell until buried.

**Table 5:** Individual medicinal plant species used for more number of ailments treatment

Names of medicinal plant species	No. of ailments treated
*Solanium incanum*	7
*Ruellia patula, Kalanchoe laciniata*, and *Croton macrostachyus*	6
*Solanum nigrum and Achyranthes aspera*	5
*Zehneria scabra*, *Lobelia gibberroa*, *Phytolacca dodecandra*, *Rumex nepalensis*, *Tragia brevipes*, and *Cucumis ficifolius*	4

Of the total collected medicinal plants species, most of them (83) were found from the wild, 20 were obtained from home gardens (those cultivated at home, where they are used also as food or purely for medicinal purposes or both), and 33 species were from both home gardens and wild habitats (Appendix: Table 10). The specific conservation sites for medicinal plant species were not established in the study area; however, the respondents listed the common locations namely Orthodox Church and Muslim Tomb forests, grazing lands, farm lands, riversides, governmental protected forests, and home gardens. Furthermore, the majority of the collected traditional medicinal plant species were herbs with 68 species followed by shrubs (40), trees (20), and climbers (8) (Appendix: Table 10).

### Informant consensus factor

Diseases in the study area are grouped into ten ailment categories and informant consensus factor (ICF) analyses were computed. Hence, febrile illness and headache scored the highest ICF value (0.59) followed by dermal diseases (0.52) (Table 6). Febrile illness was also the top recorded health problems in Gubalafto District health office. Headache was treated with *Foeniculum vulgare* and *Solanum incanum*, whereas *Carduus chamaecephalus*, *Conyza schimperi*, *Verbascum sinaiticum*, *Croton macrostachyus*, *Cynoglossum coeruleum*, *Eucalyptus globulus*, *Geranium arabicum*, *Lepidium sativum*, and *Zehneria scabra* were used for the treatment of febrile illness (Appendix: Table 10). In addition, *Ocimum lamiifolium* was used for the treatment of both headache and febrile illness (Appendix: Table 10). Two types of remedies, which were formed from mixtures of two groups of medicinal plant species, were also reported for the treatments of febrile illness. The first group of remedy was prepared from *Croton macrostachyus*, Cynoglossum *coeruleum*, *Eucalyptus globulus*, *Lepidium sativum*, and *Rumex nervosus*, and the second remedy was prepared from *Achyranthes aspera*, *Ocimum urticifolium*, *Bidens pilosa*, and *Conyza schimperi*. Parts of both groups of plant species were burnt, and fumes were inhaled for the treatments of febrile illness (Appendix: Table 10). Dermal diseases had the second ICF value and the highest number of plant species used to treat it (Table 6). The least values of ICF were found in the diseases of excretory and reproductive tracts.

**Table 6:** Disease category and their ICF values

Categories	Ailments/diseases	No. of species used	No. of use citations	ICF values
Undefined illness	Febrile and headache	29	70	0.59
Dermal	Dandruff, wound, eczema, tinia versicolor, baldness, hemorrhoid, boils/furunclosis, skin cancer, swell	66	135	0.52
Respiratory systems	Stomachache, digestion problems, bloat, diarrhea, toothache	24	41	0.43
Digestive system	Stomachache, digestion problems, bloat, diarrhea, toothache	63	103	0.39
Animal and insect cause	Cutaneous leishmaniasis, snake bite and poison, rabies, malaria, spider poison, scorpion poisons	26	40	0.36
Cultural related	Evil eye and evil spirit, diseases epidemic, general illness	16	24	0.35
Circulatory systems	Bleeding, hypertension	16	24	0.35
Musculoskeletal & nervous system	Bone broke and fracture, nerve problem	6	5	0.2
Sense organs	Eye problem, ear mites, ear bloat, trachoma, vision impairment	17	19	0.11
Excretory and reproductive	Impotency, urinary retention, expelled uterus, kidney infections, ABO-incompatible, gonorrhea, sexual diseases, retained embryo	18	19	0.06

### Informant consensus

In addition, *Ocimum lamiifolium* scored the highest number of informant consensus value (30) followed by *Eucalyptus globulus*, *Croton macrostachyus and Cynoglossum coeruleum* with 28, 24, and 21 use values, respectively. A leaf of *Ocimum lamiifolium* is drunk with coffee/tea decoction that treated headache and febrile illness. On the other hand, *Eucalyptus globulus* was used to treat common cold, appetite reduction, and febrile illness. The total informant consensus values of each medicinal plant species are given in Appendix: Table 10.

### Use values

The calculated results of use values (UV) showed that *Ocimum lamiifolium* scored the highest number, which is 0.36 and *Eucalyptus globulus* (0.33) scored higher use values than other species. Meanwhile, 40 medicinal plant species scored the least use value, which is 0.01 (Appendix: Table 10).

### Fidelity level

The fidelity level (FL) calculation was done for the most cited medicinal plant species with six and above informants. The calculation results showed that all have more than 0.5 values (Table 7). Of the results, *Rhamnus prinoides* and *Datura stramonium* scored the highest FL values, 0.97 and 0.86 respectively.

**Table 7:** FL values of the 14 most referenced medicinal plants

Species names	Primary use/s	*N*	NP	FL	Rank
*Rhamnus prinoides*	Tonsillitis	18	17	0.94	1
*Datura stramonium*	Dandruff	14	12	0.86	2
*Vicia faba*	Boils/furunclosis	6	5	0.83	3
*Cynoglossum coeruleum*	Febrile illness	21	17	0.81	4
*Ocimum latifolium*	Febrile illness	30	24	0.8	5
*Ruta chalepensis*	Stomachache	14	11	0.79	6
*Achyranthes aspera*	Tonsillitis, wound, and febrile illness	19	14	0.74	7
*Eucalyptus globules*	Febrile illness and common cold	28	19	0.68	8
*Withania somnifera*	Febrile illness, evil spirit	11	7	0.64	9
*Rumex nepalensis*	Stomachache	13	8	0.62	10
*Solanium incanum*	Stomachache	18	10	0.56	11
*Croton macrostachyus*	Febrile illness	24	12	0.5	12
*Zehneria scabra*	Febrile illness	18	9	0.5	12
*Cucumis ficifolius*	Dactylitis	10	5	0.5	12

### Preference ranking

Preference ranking values of nine medicinal plant species used to treat bleeding showed that *Achyranthes aspera* ranked first and followed by *Rumex nepalensis* (Table 8). *Achyranthes aspera* was reported to stop abnormal/excessive menstruation and much bleeding during the newborn delivery time. Informants stated that *Rumex nepalensis* stops bleeding without any patient body contact. The healers cut the leaf and soon they whispered for three times by standing at any distance from the patient by saying “stop the bleeding of the patient blood!” then drop the leaf of *Rumex nepalensis* to the ground. Immediately, the bleeding stops and the patient recover.

**Table 8:** Simple preference ranking values of nine medicinal plants used to stop bleeding in the study area

Species name	Respondents										Total	Rank
	R1	R2	R3	R4	R5	R6	R7	R8	R9	R10		
*Achyranthes aspera*	7	8	6	9	8	7	9	8	9	7	78	1
*Clutia lanceolata*	1	3	2	1	4	6	2	3	3	3	28	8
*Salila marjamic*	5	5	1	4	3	5	7	1	4	5	40	5
*Ruellia patula*	4	2	8	3	5	4	1	2	1	4	34	7
*Rumex nervosus*	3	4	3	5	6	1	3	5	5	2	37	6
*Rumex nepalensis*	6	6	9	8	7	9	6	9	6	9	75	2
*Salvia nilotica*	9	7	7	6	1	8	8	7	8	8	69	3
*Solanium incanum*	8	9	5	7	9	2	5	4	7	6	62	4
*Solanum nigrum*	2	1	4	2	2	3	4	6	2	1	27	9

### Plant parts used and mode of remedy preparation

**Fig. 2:**
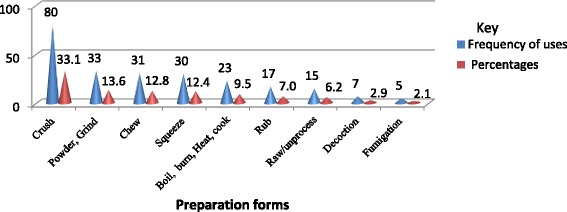
Modes of preparation of medicinal plants in Gubalafto District. The number and percentages of preparation forms of traditional plant medicines

In the study area, eight medicinal plant parts were identified for all documented remedy preparations. Among the total plant parts, 114 traditional plant remedies were prepared from the leaves of 73 medicinal plant species. Likewise, the roots of 51 medicinal plant species were used for the preparations of 76 different remedies (Table 9). Informants applied different traditional medicinal plant remedy in different ways of preparation, of which crushing was reported frequently (Fig. 2 and Appendix: Table 10). In regard to this, most of the medicinal plant remedy preparations involved the use of single plant species or a single plant part. Thus, the mixtures of different medicinal plant species or plant parts are used rarely in the traditional medicinal plant remedy preparations. In addition, the additive substances such as salt, honey, coffee, local beer, milk, butter, and SHIRO (ground legume seeds) were mixed during traditional plant medicine preparations and administrations to extract active components, to prevent the adverse effects of remedies, and to add better tastes and aromas.

**Table 9:** Plant parts and their frequency of uses for the preparation of remedies in Gubalafto District

Plant parts used	Number of reported plant species in each parts	Number of preparations in each parts
Leaf	73	114
Root	51	76
Seed	9	9
Fruit	8	9
Leaf and root	8	8
Latex	5	8
Bark	4	5
All parts	5	5
Stem and leaf	2	2
Flower, fruit and leaf; fruit, leaf and stem; root and bark	1	1

### Condition of preparations and storage techniques

The fresh and dried materials of traditional medicinal plant remedies were prepared by informants in the study area. The highest (132) number of remedies were prepared from fresh parts of medicinal plants only followed by a fewer number of traditional plant medicines (46) prepared from the dried plant parts only, and 64 remedies were prepared either from dry or fresh plant parts. Healers stored the collected traditional plant medicines in their homes for further usage mostly in powdered and raw dried forms. In this regard, clothes and plastic bags are used mainly to store the dried medicines. However, the preferences of fresh plant parts for medicine are higher than dried once.

### Route of administration, dosage determination, and taboos

**Fig. 3:**
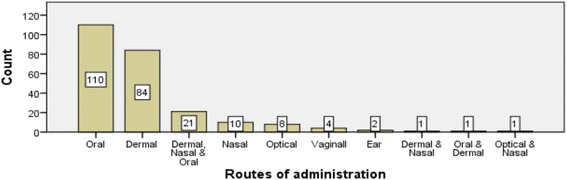
Routes of administration of traditional plant medicines. Number of traditional medicines and their administration routes reported in Gubalafto District

The respondent’s reports showed that most of the informants in the study area administered traditional plant medicines through oral and dermal routes (Fig. 3). Coffee cup, tine, finger line, teaspoon, tea glass, the number of powder droplets picked by two finger tips, and palm surface were used for dosage determinations. Medicines prepared from the plant species *Justicia schimperiana*, *Podocarpus falcatus*, *Acokanthera schimperi*, *Lobelia gibberoa*, *Euphorbia abyssinica*, *Phytolacca dodecandra*, and *Cucumis ficifolius* were reported to be toxic if overdosed. So, the informants reported that the adverse effects of toxic medicinal plant species could be alleviated by taking coffee, local beer, and flax and by eating local food like SHIRO. The healers also made different dosages of traditional medicines based on differences in gender, age, and physical condition and appearance among patients by using their experiences.

Furthermore, informants in the study area reported taboos for some medicinal plant species they used. Thus, sexual intercourse was not allowed for healers during traditional medicine preparation and offering for patients. Patients are also not allowed to have sexual intercourse at the time of using *Plantago lanceolata* medicine to treat Herpes zoster (shingles). At the time of *Tragia brevipes* prescription for treating “dactylitis”, the patients are prohibited from having sexual intercourse, eating meat, and drinking milk and coffee as well as taking modern drugs. Consequently, informants provided information that the dactylitis patients preferred traditional medicines than modern medicines. Moreover, at the time of menstruation, females are not permitted to take traditional plant medicines, nor are they allowed to touch the prepared traditional medicines for use and contacting patients who took traditional medicines. Hence, patients are kept in their houses separately until they finish the prescribed traditional plant remedies. Informants mentioned about the sources and impacts of taboos that they generated from their ancestors who did these otherwise the disease cannot be cured and the chances for relapsing were said to be high.

## Discussion

The most active participants in the study were males that performed their tasks out of their homes. Consequently, they could have chances to learn the useful values of plant species from their daily interactions. In addition, healers preferred males to transfer their indigenous medicinal plant knowledge because of their expectations that a male alone could take the plant species in far sites and forests. Similarly, the dominance of males in studies of traditional medicinal plants was also reported by other researchers [29–34]. In contrast, Friedman et al. [35] mentioned that women know more medicinal plants and these differences may be explained by cultural and occupational disparities. Furthermore, farming was the main task of the people in Gubalafto District that could provide for the higher number of farmers to develop indigenous knowledge on medicinal plants. Meanwhile, the secret transfer systems of indigenous medicinal plant knowledge for one or two individuals in the family members or friends orally at most could facilitate the disappearance of knowledge in the study area in the future. Hence, it should be shared for a considerable number of people in oral and written forms. The secret sharing styles of indigenous knowledge in the community are also performed in other places [2, 29, 30, 36–38]. On the other hand, exchanges of indigenous knowledge on medicinal plants among students in religious sites are essential for the dissemination of indigenous plant knowledge in the wider society. There are significant indigenous knowledge variations of healers and general informants showed on the number of medicinal plant species lists and associated uses they mentioned because traditional medicinal practices are the main occupations for healers in the society. In addition, the indigenous knowledge about medicinal plants from both general informants and healers is just a sequestration from generation to generation. However, the absence of significant variations between the young and elder age respondents could be that elders mostly relied on the youth for medicinal plant collections through which the younger generation got chances to know about the medicinal plant identification. This has probably helped to reduce the expected variations in indigenous knowledge on medicinal plant lists and the associated uses asked for during the study. Elders, however, gave in-depth explanations clearly about the uses of medicinal plants including that on dosages and associated histories by using their experiences than the young people.

The reason for finding a large number of documented traditional medicinal plant species and associated uses in Gubalafto District could be related to the diversity of land forms and favorable climatic conditions that the maintained varieties of plant species. Thus, the presence of different plant species in Gubalafto District could be the source of valuable indigenous knowledge used in the community. This could be related to the fact that traditional herbal medicines have helped the people to feel safe with cures indigenous to them that might also be cost-effective [1]. In addition, the preferences of the plant species like *Thalictrum rhynchocarpum*, *Ruta chalepensis*, and *Allium sativum* for the mixtures of remedy preparations with others indicated that these plant species could have high synergy potential because of their medicinal bioactive components. Likewise, the uses of *Ziziphus spina-christi* for the preservation of human corpse could be the mucilaginous substances of the smashed fresh leaves that would make the skin smooth when painted, which might reduce bacterial and fungal growths. The three documented medicinal plant species, namely, *Cirsium englerianumInula confertiflora* [23], and *Urtica simensis* [21] are found in the endemic list of plant species of Ethiopia*.*

Most of the medicinal plants were more available in the wild areas and have not been cultivated by households in the home gardens. Future efforts need to give due attention to conserve them around human habitations. Flatie et al. [39] reported that some of the medicinal plants were cultivated in home gardens for benefits other than medicine preparation. Hence, the medicinal plants are more exposed to extinction. Unless conserved, the medicinal plants may be highly eroded in the study area in the near future. Hence, the sustainable utilization of medicinal plant species should be practiced through awareness raising and conscious protection *in situ* and *ex situ*. In this regard, Balde et al. [34] stated that giving educational training for the people can help the management of traditional medicine easily. Similarly, various studies in Ethiopia [10, 40–47] and other countries [2, 9, 31, 33, 48, 49] reported the necessity of conservation and sustainable utilization of medicinal plant species in the society.

The record of the highest number of herbaceous medicinal plant species in the study could be attributed to the fact that their presence in most parts of the study area is due to the bimodal rainfall and extended availability of moisture in Gubalafto. Similarly, various studies in Ethiopia [29, 31, 39, 42, 44–46, 50] and other countries [9, 33, 51] documented the dominance of herbaceous plant species in traditional medicine preparations. In addition, Hailemariam et al. [52] stated that there were more herbaceous plant species naturally as compared to other plant habits. Works that reported dominance of woody species (shrubs and/or trees) over herbs may have been due to surveys undertaken during the dry season when most annual herbs are absent in the environment.

The presence of a higher number of plant species for traditional medicines from the family Asteraceae could be due to the adaptation potential of the species in the family in a wide range of altitudes in the study area. The evidence given for similar results from Lulekal et al. [40] revealed that the plant families that contributed to the considerably higher number of medicinal plant species were due to their wider distribution and abundance in the flora area as well as the presence of bioactive ingredients. In the same way, other ethnobotanical studies also confirmed the abundance of medicinal plant species in Asteraceae family [31, 35, 41, 49, 53, 54].

Furthermore, the ICF results of the study proved that diseases that were frequent in the study area have higher informant consensus factor (values between 0.65 and 1). In addition, the less ICF values (values between 0 and 0.65) indicated the minimal networking of indigenous people in the sharing of their knowledge on medicinal practices and this is usually the case with traditional healers. This is because of the difference in distance, altitudinal variation, and climatic conditions found among the sampled study sites in the district as well as the interest of each healer to keep his knowledge secretly from other healers for fear of piracy. The informant consensus values also indicated that the people share the knowledge of the most important medicinal plant species to treat the most frequently encountered diseases in the community. Moreover, most medicinal plant species have least use values in the study area, which could not mean that they are less effective to treat ailments. This is because the few effective medicinal plant species are reported by one or two healers. In this case, the knowledge is very secret. Likewise, the two top fidelity level value scorer medicinal plant species could be that the species found in and around home gardens which were frequently used by women to treat children diseases such as tonsillitis. In addition, more than 50% FL values of the most cited medicinal plant species suggested that there was a high level of agreement among the participants(when FL value in the arrange of 50 to 100% and less agrees when it is between 0 and 50%). Moreover, the preference ranked medicinal plant species used to stop bleeding would become therapeutic agents for emergency cases.

The greater number of traditional remedy preparations found from leaf parts of medicinal plant species had a better accessibility during field collection, ease of preparation, and effectiveness due to bioactive components in their parts. In the same way, the detailed reason for leaves as the most potential sources of traditional remedy preparation was suggested by various studies [9, 31, 32, 34, 35, 43, 44, 50, 51, 55, 56]. In addition, the uses of leaves have been supported by most investigations than roots, since using roots leads to the disappearance of the medicinal plant species forever [2, 3, 10, 30, 31, 34, 40, 46, 48, 54, 57, 58]. In the same ways, the fresh parts of medicinal plant species were the most preferred in remedy preparation due to its availability in the area at the time of necessity. In addition, the traditional medicines prepared from fresh parts of medicinal plant species helped to keep their efficacy and safety for immediate uses. On the other hand, medicinal plant remedy used in dried form could reduce its potential efficacy and safety due to the bioactive components exposed to evaporation, other chemical reactions, and decay with available moisture in storage. In this regard, other researchers also documented the preference of fresh medicine for uses [42, 44–46, 50, 53].

In the same vein, most of the administered remedies via oral routes indicated the higher prevalence of internal ailments in the study area. However, the dose should be given in great care in the oral system than in the dermal since it might cause other severe internal problems. Similarly, various research findings mentioned oral application as the primary route of administration in traditional plant medicines [1, 43–45, 50, 53, 55, 58–60]. Giday et al. [61] in their studies in southwest Ethiopia reported that most bench herbal remedies were applied topically on the skin. The additives mixed during remedy preparations and administrations could dilute the crude plant medicines and make it safer for the patient. Likewise, the significances of additives in the preparations of traditional plant medicines were described by other research results [30, 36, 44, 55]. The lack of standardized measuring units for traditional medicines and its drawbacks have been mentioned by many researchers [2, 30, 37, 40]. Moreover, the reports on side effects related to inappropriate dosages might reduce the traditional uses of plants for healthcare systems by the people.

### Comparative review results from similar studies

All documented traditional medicinal plant species in this study were searched in the published similar research works in Ethiopia in particular and the world outside Ethiopia [1–3, 9, 10, 31–36, 38, 40, 42, 45–51, 53, 55–84]. From this review, 121 recorded medicinal plant species in this study are likely also to be found in other parts of Ethiopia and other countries. Among these, 73 of the species had similar uses with other studies and 49 had unlike functions (Appendix: Table 10 & 11) in the medical lore of Gubalafto. In addition, the medicinal plants, which have similar uses within this study, also showed different preparation and application techniques. Furthermore, 17.85 mean percentages of new uses were found from the comparative reviews (Appendix: Table 11). Hence, doing an ethnobotanical study on the traditional uses of medicinal plant species in different areas would help to document new additional uses from already known traditional medicinal plant species. On the other hand, 14 medicinal plant species documented in this study were not found in any of the related literature reviewed (Appendix: Table 10). These medicinal plants are *Actiniopteris dimorpha*, *Aloe weloensis*, *Conyza schimperi*, *Grewia kakothamnos*, *Haplocarpha rueppelii, Huernia macrocarpa*, *Indigofera brevicalyx*, *Kedrostis gijef*, *Melilotus suaveolens*, *Oncocalyx schimperi*, *Polygala sphenoptera*, *Pteris dentata*, *Ranunculus stagnalis*, and *Thesium kilimandscharicum.*

## Conclusions

The traditional medicinal plant species are the potential sources in traditional healthcare systems of the people in Gubalafto District. The study confirmed that the people have been using medicinal plant species and the associated indigenous knowledge as a primary option although modern health services are expanding in the District. The documented new traditional medicinal plant species in this study have validated the call for further scientific research on their ethnobotany and other aspects. The study also ensured that the communities did not have turned deep insight in the conservation of the useful medicinal plant species and associated uses. The indigenous knowledge tied with medicinal plant species were found in both younger and older age groups by which the younger reduced their interest in searching, sharing, and documenting processes. Higher use value, preference ranking scores, and fidelity level values of the recorded medicinal plant species would empower the future pharmaceutical and phytochemical investigations and conservation practices. In this connection, attention should be drawn to the conservations of traditional medicinal plants and associated indigenous knowledge in the study area to sustain them in the future.

## Appendix

**Table 10:** List of human traditional medicinal plant species, family and vernacular names of the species, number of use reports, use value, ailments treated, plant parts used, condition of preparations, route of administrations, methods of preparations and applications, habit in Gubalafto Districts, Ethiopia

Sample no.	Family names	Scientific names	Local names	Ha	No. of use report	Use of value (UV)	Ailment treated	PU	Cp	Ra	Methods of preparation and applications	Comparison with similar studies	Ht	CN
1.	Acanthaceae	*Barleria eranthemoides* R. Br. ex C. B. Clarke	Setaf/senkolla	H	6	0.07	Wound	L	F	D	Crush and tie	32^▼^, 50^▼^, 40^▼^	W	GC180
							Visual impairment	R	F	Op	Paint the ash together with butter			
		*Justicia schimperiana* (Hochst. ex Nees) T. Anders.	Sensel	S	5	0.06	Typhoid and malaria	L	F	O	Crush and squeeze then drink with coffee	31^▲^, 38^▼^, 54^▼^, 37^▼^, 50^▲^, 59^▼^, 76^▼^, 68^▼^, 63^▲^, 46^▲^, 65^▼^, 55^▲^, 32^▼^, 70^▼^, 73^▼^, 81^▲^, 83^▼^	H	GC154
							Liver problem	L	F	O	Squeeze then drink			
		*Ruellia patula* Jacq.	Duaduatie/goregondie	H	4	0.05	Baldness	Ft	D	D	Use ash to paint together with butter	71^▼^, 75^▲^	W	GC225
							Bleeding and wound	L	F	D	Crush and tie			
							Stomach problem	R	F/D	O	Crush and smash in water for 3 days then drink			
							Snake bite	R	F	O	Chew and swallow the juice			
2.	Actiniopteridiacae	*Actiniopteris dimorpha* Pic. Serm.	Esat adrik	H	2	0.02	Fire burn	All	F/D	D	Paint the ash/crush and wash with juice	–	W	GC211
	Alliaceae	*Allium sativum* L.	Nech shinkurt	H	11	0.13	Evil eye	Ft	F	N	Crush then sniff	68^▲^, 31^▼^, 37^▼^, 63^▲^, 50^▼^, 36^▼^, 29^▲^, 46^▼^, 78^▲^, 65^▼^, 67^▲^, 80^▼^, 42^▼^, 45^▲^, 70^▼^, 72^▼^, 59^▲^, 77^▼^, 55^▲^, 38^▲^, 73^▼^, 47^▼^, 82^▼^, 83^▼^	H	GC011
							Stomachache	L	F	O	Chew and swallow			
3.	Aloaceae	*Aloe weloensis* Sebsebe	Eret tafa	H	4	0.05	Wound	Lx	F	D	Paint	–	WH	GC210
							Malaria	Lx	F	O	Isolate and drink			
							ABO incompatibility	R	D	O	Powderize and mix with honey then drink			
4.	Amaranthaceae	*Achyranthes aspera* L.	Telenge	H	19	0.23	Wound	L	F	D	Pound and tie	2^▲^, 31^▲^, 49^▼^, 63^▲^, 50^▼^, 78^▲^, 46^▼^, 40^▼^, 65^▼^, 66^▼^, 67^▼^, 45^▼^, 48^▼^, 70^▼^, 71^▲^, 73^▲^, 59^▼^, 38^▼^, 82^▲^, 83^▼^, 84^▲^	WH	GC025
							Fever	L	F	DNO	Boil the concoction then fumigate the fume			
							Eye dusts and ear mites	L	F	Op, N	Squeeze the concoction then drop using cotton			
							Tonsillitis	L	F	O	Chew and absorb the juice with coffee			
							Excessive bleeding after birth	L	F	Vl	Rub and insert then cover with cloth			
5.	Anacardaceae	*Rhus retinorrhoea* Oliv.	Talo enbis	T	1	0.01	Tonsillitis	L	F	O	Squeeze then drink	50^▼^, 78^▼^	W	GC187
							Stomachache	R	D	O	Crush and smash in water for 3 days then drink the filtrate with honey			
		*Schinus molle* L*.*	Kundoberberie	T	2	0.02	Common cold	L	F	N	Rub and sniff	31^▼^, 38^▼^, 63^▲^, 78^▼^, 77^▼^, 46^▼^, 70^▼^, 73^▼^, 47^▼^	H	GC155
6.	Apiaceae	*Agrocharis melanantha* Hochst.	–	H	1	0.01	Stomachache	R	F	O	Chew and absorb the juice	29^▼^	W	GC209
		*Ferula communis* L.	Dog	H	2	0.02	Impotency	R	F/D	O	Powderize the concoction then drink with Tella	78^▼^, 59^▼^, 63^▲^, 46^▼^	W	GC072
		*Foeniculum vulgare* Mill.	Ensilal	H	4	0.05	Headache	R	F	O	Crush, mash, and filtrate then drink	31^▲^, 38^▼^, 54^▲^, 50^▼^, 42^▲^, 63^▼^, 68^▲^, 46^▼^, 65^▼^, 70^▼^, 80^▼^, 35^▲^, 82^▼^, 83^▲^	WH	GC137
							Tooth and stomachache	R	D	O	Crush, mash, boil, and filter then drink			
							Kidney infection	L	F	O	Decoct and filter then drink			
7.	Apocynaceae	*Acokanthera schimperi* (A.DC.) Schweinf.	Miriez	S	1	0.01	Wound	L	D	D	Crush then paint	32^▼^, 63^▼^, 50^▼^, 40^▼^, 65^▼^, 45^▼^, 59^▲^, 77^▼^, 79^▼^, 38^▲^, 73^▼^, 81^▼^	W	GC047
		*Carissa spinarum* L.	Agam	S	7	0.08	Snake poison	L	F	O	Chew and swallow	31^▲^, 38^▼^, 50^▲^, 37^▲^, 59^▲^, 76^▼^, 63^▼^, 61^▼^, 68^▼^, 65^▲^, 29^▲^, 46^▼^, 45^▼^, 70^▼^, 79^▼^, 40^▲^, 78^▼^, 73^▲^, 47^▲^, 81^▲^, 82^▼^, 83^▲^	W	GC021
							Evil spirit	R	D	N	Crush and fumigate			
							Stomachache	R	F/D	O	Crush, mash, and filter then drink			
8.	Arecaceae	*Phoenix reclinata* Jacq.	Sensel	S	3	0.04	Sexual diseases	R	D	O	Powderize the concoction then drink with honey and butter	68^▼^, 77^▼^	WH	GC201
9.	Asclepiadaceae	*Calotropis procera (*Ait.) Ait.f.	Tobia	S	4	0.05	Eczema	Lx	F	D	Paint	38^▲^, 78^▼^, 9^▼^, 75^▼▲^, 33^▼^, 36^▼^, 51^▼^, 72^▲^, 63^▲^, 77^▼^, 66^▲^, 58^▲^, 73^▼^, 82^▲^, 83^▼^, 84^▼^	W	GC035
							Hemorrhoid	Lx	F	D	Paint			
		*Huernia macrocarpa* (A.Rich) Sprenger	Yemidir kulkual	H	1	0.01	Skin cancer	Lx	F	D	Mix with Sumanfar then insert at the spot	–	W	GC100
10.	Asteraceae	*Artemisia absinthium* L.	Natra	H	5	0.06	Evil eye	L	F	N	Rub and sniff	68^▼^, 29^▲^, 46^▼^, 35^▼^, 73^▲^	H	GC176
							Tonsillitis	L	F	O	Squeeze the concoction then drink			
		*Artemisia afra* Jack. ex Willd.	Chikugn	H	5	0.06	Evil eye	All	F	N	Sniff	68^▼^, 63^▲^, 50^▲^, 42^▼^, 45^▼^, 70^▲^, 76^▼^, 48^▼^, 38^▲^, 84^▼^	H	GC168
		*Bidens pilosa* L.	Yeseytan merfie	H	2	0.02	Evil spirit	All	F/D	DNO	Fumigate	53^▼^, 72^▼^, 29^▼^, 46^▲^, 70^▼^	W	GC184
		*Carduus chamaecephalus* (Vatke.) Oliv. & Hiern	Kushele/dendero	H	7	0.08	Febrile illness	R	F/D	DNO	Crush and mash then paint body with extract/decoct then fumigate	68^▼^	W	GC197
		*Cirsium englerianum* O. Hoffm.	Kushele	H	1	0.01	Intestinal parasite	R	D	O	Powderize the concoction and drink with honey. After a few minutes, drink local beer (Tella)	63^▼^	W	GC050
		*Conyza schimperi* Sch. Bip. ex A. Rich	Wereza ktel	H	2	0.02	Febrile illness	All	F	DNO	Decoct the concoction and fumigate	–	WH	GC215
		*Guizotia abyssinica* (L.f.) Cass.	Nug	H	1	0.01	Gastric	Sd	D	O	Chew and swallow	37^▼^, 65^▼^	W	GC226
		*Haplocarpha rueppelii* (sch.Bip.) Beauv.	Getn	H	1	0.01	Bleeding	L	F	D	Rub and tie	–	W	GC232
		*Inula confertiflora* A. Rich.	Weinagift	S	3	0.04	Toothache	L	F	O	Take into the mouth, chew ,for a few minutes, and spit then drink coffee	46^▼^, 65^▼^	WH	GC220
							Tonsillitis	L	F	O	Squeeze then drink			
		*Kleinia abyssinica* (A. Rich.) A. Berger	Este-maza	H	3	0.04	Brain weakness	R	F/D	O	Crush the concoction until powderized, mix honey, boil to distillate, and drink	79^▼^, 40^▼^	W	GC190
		*Vernonia amygdalina* Del. in Caill*.*	Grawa/merari kelewa	S	2	0.02	Gout	L	F	DNO	Fumigate	31^▼^, 38^▼^, 79^▼^, 78^▼^, 37^▼^, 50^▼^, 36^▼^, 54^▼^, 40^▼^, 72^▼^, 42^▼^, 76^▼^, 63^▼^, 61^▼^, 68^▼^, 29^▼^, 45^▼^, 70^▼^, 65^▼^, 81^▼^, 82^▼^, 83^▼^	WH	GC055
11.	Balanitaceae	*Balanites aegyptiaca* (L.) Del.	Bedena	T	3	0.04	Dandruff	L	F	D	Crush the concoction then paint	32^▼^, 40^▼^, 70^▼^, 75^▼^, 83^▼^	WH	GC240
							Stomachache	L	F	O	Chew and absorb the juice			
12.	Boraginaceae	*Cordia africana* Lam.	Wanza	T	1	0.01	Eczema	L	F/D	D	Paint the concoction ash together with butter	31^▲^, 38^▼^, 78^▼^, 30^▼^, 48^▼^, 54^▼^, 42^▼^, 63^▼^, 68^▼^, 40^▼^, 77^▼^, 45^▼^, 70^▼^, 73^▼^, 47^▼^	WH	GC133
		*Cynoglossum coeruleum* Hochst. ex A. DC.	Chugagot	H	21	0.25	Febrile illness	L	F	DNO	Decoct the concoction then fumigate	31^▲^, 38^▲^, 63^▲^, 47^▲^, 81^▲^	W	GC114
							Eye problem	L	F/D	N	Crush concoction then insert in the nasal tube			
							Febrile illness	L	F	D, N	Squeeze then paint the body and drink with honey			
		*Heliotropium cinerascens* D.C*.*	Nechilo	H	5	0.06	Wound	L	F	D	Crush and tie	83^▼^	W	GC199
							Eye mites	L	F	Op	Crush the concoction*,* squeeze, filtrate, and insert			
13.	Brassicaceae	*Brassica oleracea* L.	Tikle gomen	H	1	0.01	Gastric	L	D	O	Powderize, mix honey for 2 days then eat	49^▼^	H	GC191
		*Lepidium sativum* L.	Fetto	H	2	0.02	Febrile illness	Sd	D	DNO	Burn the concoction and fumigate	31^▼^, 38^▼^, 54^▲^, 50^▼^, 42^▼^, 68^▼^, 77^▼^, 29^▼^, 65^▲^, 45^▼^, 55^▼^, 78^▼^, 73^▲^, 65^▼^, 81^▼^, 84^▼^	H	GC213
14.	Capparidaceae	*Boscia salicifolia* Olive.	Tisha	T	2	0.02	Ear mites	L	F	O	Squeeze and mix lemon juice then drink	40^▼^, 33^▲^, 73^▼^	W	GC179
							Eye problem	L	F	Op	Squeeze and filtrate then insert			
		*Capparis tomentosa* Lam.	Gimero	Cl	3	0.04	Evil eye and evil spirit	R	F/D	DNO	Crush then fumigate	62^▼^, 38^▲^, 50^▲^, 53^▼^, 37^▼^, 59^▲^, 63^▲^, 77^▲^, 46^▼^, 40^▼^, 55^▲^, 70^▼^, 78^▼^, 73^▲^, 47^▼^, 81^▲^, 83^▼^	W	GC023
15.	Caricaceae	*Carica papaya* L.	Papya	T	1	0.01	Intestinal parasite	Sd	D	O	Powderize then eat with honey	49^▼^, 1^▼^, 40^▼^, 38^▼^, 53^▼^, 65^▼^, 36^▼^, 72^▼^, 30^▼^42^▲^, 59^▼^, 63^▼^, 68^▼^, 77^▼^, 58^▼^, 69^▼^, 70^▲^, 78^▲^, 73^▼^, 83^▲^	H	GC098
16.	Celastraceae	*Catha edulis* (Vahl) Forssk. ex Endl.	Chat	S	1	0.01	Intestinal parasite	L	F	O	Decoct then drink the extract	48^▼^, 54^▼^, 37^▲^, 68^▼^, 65^▼^, 46^▼^, 40^▼^, 45^▼^, 55^▼^, 70^▼^, 78^▼^, 47^▼^	H	GC196
17.	Chenopodiaceae	*Chenopodium murale* L.	Amedmado	H	1	0.01	Cutaneous leishmaniasis	R,L	D	D	Point the concoction ash	38^▼^, 51^▼^, 63^▼^, 73^▲^	W	GC136
18.	Crassulaceae	*Kalanchoe laciniata* (L.) DC.	Endahula/shilkak	H	2	0.022	Swelling	L	F	D	Heat and apply on the spot	37^▲^, 63^▼^, 40^▼^	W	GC084
							Broken bone	L	F	O	Heat and rub the target part			
							Expelled uterus	L	F	Vl	Crush and pushed inside			
							Hip obesity	R	F	D	Crush the concoction then paint and rub			
							Tonsillitis	R	F	N	Peal and chew or sniff			
							Toothache	R	F	O	Take into the mouth, chew for a few minutes then spitted out and drink coffee			
19.	Cucurbitaceae	*Cucumis ficifolius* A. Rich.	Yemdir enboy	H	11	0.13	Dactylitis	Ft	F	D	Make a hole and insert it	31^▼^, 38^▼^, 79^▼^, 37^▲^, 50^▼^, 78^▼^, 59^▲^, 63^▲^, 46^▲^, 40^▲^, 65^▲^, 70^▼^, 73^▼^, 81^▲^, 82^▼^	W	GC139
							Hemorrhoids	R	F/D	D	Crush, mix with honey then tie			
							Rabies	R	D	O	Powderize then drink with water and milk			
							Stomachache	R	F	O	Chew and absorb the juice			
		*Cucurbita pepo* L.	Duba	Cl	1	0.01	Excessive bleeding after birth and menstruation	R	D	O	Powderize and drink with water	31^▼^, 38^▼^, 50^▼^, 48^▼^, 37^▼^, 63^▼^, 77^▼^, 29^▼^, 70^▼^, 65^▼^, 78^▼^, 83^▼^	H	GC166
							Swelling	R,L	F	D	Roast then paint and tie			
		*Kedrostis gijef* (Forssk.)C. Jeffrey	Ergobergo	Cl	1	0.01	Swelling	L	F/D	D	Crush and powderize then paint	–	W	GC228
		*Zehneria scabra* (Linn. f.) Sond..	Hareg resa/ harresa	H	18	0.21	Febrile illness	L	F	DNO	Squeeze the concoction then cream	38^▲^, 50^▼^, 78^▲^, 53^▼^, 37^▲^, 63^▲^, 46^▼^, 68^▲^, 65^▼^, 81^▼^, 84^▼^	WH	GC149
							Wound	L	F	D	Powderise then cream			
							Febrile illness	L	F	DNO	Boil and fumigate			
							Liver problem	L	F	O	Squeeze then drink with water			
20.	Ebenaceae	*Euclea racemosa* Murr.	Dedeho	S	1	0.01	Dysuria	L	F	O	Squeeze then drink with honey	63^▼^, 50^▼^, 46^▼^, 32^▼^, 79^▼^, 73^▼^, 83^▼^	W	GC018
							Dandruff	R	F/D	D	Paint the ash together with butter			
21.	Euphorbiaceae	*Clutia lanceolata* Forssk.	Fiyelefej	S	4	0.05	Evil eye	R	F/D	D	Crush the concoction then tie	31^▼^, 63^▼^	W	GC135
							Excessive bleeding after birth	R	F	O	Crush, mash for 3 days, and filtrate then drink			
		*Croton macrostachyus* Del.	Mekanisa	T	24	0.29	Liver problem	B	F/D	O	Powderize the concoction then drink with honey	31^▲^, 40^▼^, 61^▼^, 68^▲^, 77^▼^, 59^▲^, 76^▲^, 63^▲^, 57^▲^, 48^▼^, 54^▲^, 37^▲^, 42^▲^, 29^▲^, 46^▲^, 45^▲^, 55^▲^, 70^▲^, 79^▲^, 65^▲^, 38^▲^, 78^▲^, 47^▼^, 81^▲^, 82^▲^, 83^▲^	W	GC130
							Stomachache	B	D	O	Crush and eat with honey			
							Gonorrhea	B	F	O	Squeeze then drink. After a few minutes, eat hen heart or drink Tella			
							Malaria	Ft	F	O	Crush and mash then drink with Tella			
							Atopic dermatitis	L	F	D	Squeeze the sap then paint			
							Epidemic	L	F/D	DNO	Burn and fumigate			
							Febrile illness	L	F/D	DNO	Crush, decoct, and fumigate			
		*Euphorbia abyssinica* Gmel.	Kulkual	T	3	0.04	Take out spine	Lx	F	D	Take the latex then paint on the spot	50^▼^, 59^▼^, 63^▼^, 78^▼^, 46^▼^, 45^▲^, 73^▼^, 81^▼^	WH	GC164
							Stomach problem	Lx	F	O	Take the latex, bake with bread then eat			
		*Euphorbia tirucalii* L*.*	Kinchib	S	2	0.02	Tinea versicolor	Lx	F	D	Take the latex then paint	54^▼^, 37^▼^, 50^▼^, 42^▼^, 59^▲^, 77^▼^, 63^▲^, 29^▼^, 46^▼^, 69^▼^, 35^▲^, 73^▼^, 47^▼^, 83^▼^	WH	GC131
							Wound	R	D	D	Powderize then paint			
		*Ricinus communis* L.	Chakima/ Gulo	S	2	0.02	Ear mites	L	F	Er	Squeeze and insert	31^▼^, 38^▼^, 79^▼^, 50^▼^, 9^▼^, 57^▼^, 49^▼^, 40^▼^, 48^▼^, 54^▼^, 72^▼^, 42^▼^, 68^▼^, 77^▼^, 29^▼^, 46^▼^, 55^▼^, 67^▼^, 80^▼^, 66^▼^, 65^▼^, 70^▼^, 78^▼^, 59^▼^, 73^▼^, 47^▼^, 81^▲^, 83^▼^, 84^▼^	H	GC170
							Stomachache	R	F/D	DNO	Fumigate			
		*Tragia brevipes* Pax.	Awulalit	H	7	0.08	Dactylitis	R	D	D	Crush, powderize, and add butter then tie	63^▼^	W	GC013
							Impotency	R	F/D	O	Chew and absorb the juice	63^▲^, 40^▼^		
							Stomachache	R	F	O	Chew and absorb the juice			
							Retained placenta	R	F	Vl	Crush and insert in a cloth through the vaginal tube			
22.	Fabaceae	*Acacia seyal* Del.	Duret	T	7	0.08	Dandruff	L	F	D	Crush and paint with water	32^▼^, 46^▼^, 53^▼^, 40^▼^, 45^▼^, 70^▲^	WH	GC229
							Wound	L	F	D	Chew and tie/cream			
							Swelling	L	F	D	Crush and tie			
		*Calpurnia aurea* (Ait.) Benth.	Digita	S	1	0.01	Excessive bleeding after birth	R	F/D	O	Crush or powderize the concoction then drink with water	38^▼^, 31^▼^, 37^▼^, 50^▼^, 59^▼^, 76^▼^, 63^▲^, 68^▼^, 77^▼^, 29^▼^, 46^▼^, 55^▼^, 70^▼^, 40^▼^, 65^▼^, 73^▼^, 81^▼^, 83^▲^	WH	GC020
		*Cicer arietinum* L.	Shinbira	H	1	0.01	Malaria	Sd	D	O	Germinate then eat the concoction.	63^▲^, 68^▼^	WH	GC115
		*Indigofera brevicalyx* Bak.f	–	H	3	0.04	Snake poison	R	F	O	Chew and absorb the juice	–	W	GC221
		*Indigofera spicata* Forssk.	–	H	4	0.05	Snake bite and poison	R	F	O	Chew and absorb the juice	50^▼^, 61^▼^, 68^▲^, 29^▼^, 40^▼^, 45^▲^, 82^▼^	W	GC205
		*Melilotus suaveolens* Ledeb.	Egug	H	2	0.02	Herpes zoster	L	D	O	Crush and drink with water	–	W	GC186
		*Senna septemtrionalis* (Viv.) Irwin & Barneby	–	S	1	0.01	Cough, lung cancer, and brain problem	L	F	D	Crush, smash in water, filter then drink	40^▼^	W	GC189
		*Trigonella foenum-graecum* L.	Abish	H	1	0.01	Gastric	Sd	D	O	Grind, mix with water, and drink	38^▲^, 78^▲^, 73^▲^	WH	GC181
		*Vicia faba* L.	Bakela	H	6	0.06	Boils/furunclosis	Sd	D	D	Crush with teeth then paint	63^▼^, 80^▼^, 47^▼^, 84^▲^	WH	GC109
							Nerve problem	Sd	D	D	Grind and mix butter then paint and stay on sunlight			
23.	Geraniaceae	*Geranium arabicum* Forssk.	Mencherer	H	7	0.08	Febrile illness	R	D	DNO	Crush the concoction, decoct, and use fume to fumigate	50^▼^, 29^▲^	W	GC203
							Stomachache	R	F	O	Crush, decoct, and filter then drink			
24.	Gingibraceae	*Zingiber officinale* Rosc.	Gingible	H	5	0.06	Stomachache	R	F	O	Chew and swallow the juice/drink with tea	38^▼^, 36^▼^, 72^▼^, 37^▼^, 42^▲^, 61^▼^, 68^▲^, 77^▲^, 45^▲^, 67^▲^, 58^▼^, 74^▼^, 70^▼^, 65^▼^, 78^▲^, 81^▲^, 84^▼^	H	GC231
							Swelling	L	F	D	Chew and tie			
25.	Hypericaceae	*Hypericum quartinianum* A.Rich	Amujia	S	2	0.02	Eczema	R	F/D	D	Crush and mix with 1-year stayed ash and camel’s feces then paint with butter	50^▼^, 46^▼^	W	GC224
							Stomachache	R,L	F/D	O	Crush and immerse in water then drink			
26.	Lamiaceae	*Ajuga integrifolia* Buch.-Ham. ex D.Don	Tut astil	H	2	0.02	Tonsillitis	L	F	O	Rub and squeeze then drink	46^▼^, 45^▼^, 70^▼^, 73^▼^	W	GC214
		*Clerodendrum myricoides* (Hochst.) Vatke	Misroch	S	3	0.04	Wound	L	F/D	D	Crush the concoction and tie/rub and tie alone	31^▼^, 38^▼^, 50^▼^, 3^▼^, 53^▼^, 48^▼^, 76^▼^, 63^▼^, 61^▼^, 68^▼^, 77^▼^29^▼^, 46^▲^, 40^▼^, 70^▼^, 78^▼^, 73^▼^, 83^▼^	W	GC016
		*Leonotis ocymifolia* (Burm.f.) Iwarsson	Ferezeng	S	5	0.06	Brain problem	L	F/D	O	Crush the concoction and immerse in water then drink with honey	38^▼^, 50^▼^, 78^▼^	W	GC192
							Swelling	L	D	O	Crush until powderized then drink with water			
		*Meriandra dianthera* (Roth ex Roem & schult.) Briq.	Mentese	S	3	0.04	Trachoma	Fr	D	Op	Burn the concoction and paint the eye with the ash	38^▼^	WH	GC194
		*Ocimum lamiifolium* Hochst. ex Benth.	Dama kesie/alemsela	S	30	0.36	Headache, febrile illness	L	F	O	Cook and drink with coffee	31^▲^, 38^▲^, 79^▼^, 57^▼^, 54^▼^, 50^▲^, 42^▼^, 59^▲^, 76^▼^, 63^▲^, 61^▼^, 68^▼^, 29^▼^, 46^▲^, 45^▲^, 70^▲^, 65^▲^, 78^▼^, 81^▲^, 83^▲^, 84^▲^	WH	GC129
							Febrile illness	L	F	DNO	Burn the concoction and fumigate			
		*Plectranthus cylinderaceus* Hochst. ex. Benth	Tsezeteza	H	6	0.06	Stomach problems	L	F	O	Squeeze then drink	29^▼^, 32^▼^	WH	GC206
		*Plectranthus lanuginosus* (Hochst. ex Benth.) Agnew	Aguacher	H	3	0.04	Expel dusts in the eye	L	F	Op	Rub and drop the fluid then cover with cotton	–	W	GC216
		*Salvia merjamie* Forssk.	Shehara kitel	H	1	0.01	Bleeding	L	F	D	Rub and tie/paint	31^▼^	WH	GC204
		*Salvia nilotica* Jacq.	–	H	2	0.02	Bleeding	L	F	D	Squeeze then tie	68^▲^, 50^▼^, 46^▼^, 45^▼^	W	GC233
							Ear mites	L	F	Er	Squeeze then cover with cotton			
27.	Lobeliaceae	*Lobelia gibberoa* Hemsl.	Jibara	T	3	0.04	Eye problem	R	F/D	O	Crush and immerse in water then drink		W	GC119
							Impotency	R	F/D	O	Crush then mix with coffee and drink			
							Malaria	R	F/D	O	Crush and powderize then drink with water			
							Epilepsy	Sd	D	O	Give with Teff injera			
28.	Loranthaceae	*Englerina woodfordioides* (Schweinf.) Balle	Yekinchib teketila	H	4	0.05	Wound	L	D	D	Powder then paint	55^▼^	WH	GC200
		*Oncocalyx schimperi* (A.Rich.)M Gilbert	Yebedena teketila	H	1	0.01	Dactylitis	L	F	D	Chew the concoction then tie	–	W	GC202
29.	Malavaceae	*Sida tenuicarpa* Vollesen	Chifrg	H	2	0.02	Eye problem	R	F	Op	Crush the concoction, squeeze, and insert	63^▼^	W	GC153
		*Gossypium barbadense* L.	Tit	S	1	0.01	Eczema	Sd	D	D	Mix ash with honey then tie	63^▼^, 29^▼^	H	GC096
30.	Melianthaceae	*Bersama abyssinica* Fresen.	Azamir	S	2	0.02	Rabies	R and L	F/D	O	Crush the concoction then drink with milk	78^▼^, 48^▼^, 54^▼^, 37^▼^, 40^▼^, 65^▼^, 42^▼^, 63^▼^, 29^▼^, 31^▼^, 70^▼^	W	GC107
31.	Menispermaceae	*Stephania abyssinica* (Dillon & A. Rich.) Walp.	–	H	4	0.05	Intestinal parasite	R	D	O	Powderize the concoction then drink with honey. After a few minutes, drink Tella	31^▼^, 59^▼^, 63^▼^, 50^▼^, 78^▼^, 68^▼^, 40^▼^65^▼^, 46^▼^, 47^▼^	W	GC121
32.	Mersinaceae	*Myrsine africana* L.	Kechemo	S	1	0.01	Stomachache	R and L	F/D	O	Crush, immerse in water, and mix with butter and honey then drink	50^▼^, 78^▼^, 76^▼^, 29^▼^, 46^▼^, 40^▼^, 65^▼^, 55^▼^	W	GC217
33.	Moraceae	*Ficus carica* L.	Beles	S	3	0.04	Eczema	L	F/D	D	Powder paint with butter/tie	31^▼^, 63^▼^, 80^▲^	W	GC104
		*Ficus sur* Forssk.	Sholla	T	2	0.02	Cutaneous leishmaniasis	R,L	D	D	Powderize the concoction then paint with honey	1^▼^, 31^▼^, 48^▼^, 63^▼^, 46^▲^	W	GC090
		*Ficus vasta* Forssk.	Warka	T	2	0.02	Stomachache	R	F	O	Chew and absorb the juice	31^▼^, 63^▼^, 68^▼^	W	GC162
34.	Moringaceae	*Moringa stenopetala* (Bak.f.) Cuf.	Shiferaw	T	2	0.02	Hypertension	L	F	O	Squeeze then drink/crush then boil, filtrate, and drink	54^▼^, 68^▲^, 29^▼^, 70^▲^	WH	GC178
35.	Myricaceae	*Myrica salicifolia* A. Rich.	Shinet	T	2	0.02	Evil eye and evil spirit	B	D	N	Crush powder then sniff with the nose	63^▼^, 65^▼^, 46^▼^	W	GC106
							Liver problem	B	F/D	O	Crush the concoction and drink with honey			
							Joint pain	Rb	D	O	Crush and powderize then drink with honey			
36.	Myrtaceae	*Eucalyptus globulus* Labill.	Nech bahirzaf	T	28	0.33	Febrile illness	L	F	DNO	Decoction and fumigate	31^▲^, 38^▲^, 50^▲^, 53^▼^, 54^▲^, 37^▲^, 42^▲^, 63^▲^, 68^▼^, 77^▼^, 46^▲^, 55^▼^, 67^▲^, 80^▼^, 65^▼^, 70^▼^, 78^▼^, 73^▲^, 83^▲^, 84^▲^	WH	GC167
							Common cold	L	F	DNO	Decoction then fumigate			
							Appetite	L	D	O	Powderize the concoction and drink the decoction			
37.	Olacaceae	*Ximenia americana* L..	Enkoy	S	3	0.04	Swelling	R and L	F	O, D	Chew and swallow the leaf/crush the root and tie	63^▼^, 77^▼^, 40^▼^, 75^▼^, 47^▼^, 81^▲^, 83^▼^	W	GC054
							Stomachache	R	F	O	Powder ize then drink with water			
38.	Oleaceae	*Jasminum abyssinicum* Hochest. ex DC.	Tenbelel	Cl	3	0.04	Eye problem	L	F	Op	Crush and squeeze the concoction then insert with cotton	63^▼^, 65^▼^, 77^▼^	W	GC012
		*Olea europaea subsp. cuspidata* L.	Woira	T	1	0.01	Dandruff	R	F/D	D	Crush and paint the concoction/crush and paint alone	31^▼^, 38^▼^, 50^▼^, 53^▼^, 34^▼^, 40^▼^, 48^▼^, 63^▼^, 46^▼^, 45^▲^, 80^▼^, 75^▼^, 70^▼^, 65^▼^, 47^▼^, 81^▼^	W	GC079
39.	Oliniaceae	*Olinia rochetiana* A. Juss.	–	T	1	0.01	Eczema	L	F	D	Crush and tie	50^▼^, 48^▼^, 46^▼^, 40^▼^, 65^▼^, 70^▼^	WH	GC219
40.	Oxalidaceae	*Oxalis corniculata* L*.*	Yebere chew	H	1	0.01	Tinea versicolor	L	F	D	Rub until recovery	70^▼^, 71^▼^	W	GC227
41.	Phytolaccaceae	*Phytolacca dodecandra* L’Her.	Mehan Endod	Cl	3	0.04	Rabies	R	F/D	O	Grind then drink with water	31^▲^, 38^▲^, 63^▲^, 50^▼^, 78^▼^, 61^▲^, 68^▲^, 77^▲^, 40^▼^, 42^▲^, 59^▼^, 48^▲^, 54^▲^, 37^▼^, 29^▼^, 46^▼^, 55^▼^, 70^▼^, 65^▼^, 47^▲^, 81^▲^	W	GC024
							Ascariasis	R	F/D	O	Crush the concoction, mix water then drink			
							Malaria	R	F	O	Crush the concoction and drink with water			
							Liver problem	R	F	O	Crush the concoction, mix water, filtrate then drink			
							Prevent snake poison	L	F	O	Squeeze then drink with honey			
42.	Plantaginaceae	*Plantago lanceolata* L.	–	H	3	0.04	Spider poison	L	F	D	Powderize then paint together with butter	31^▼^, 38^▼^, 63^▼^, 50^▼^, 61^▼^, 77^▼^, 46^▼^, 65^▼^, 55^▲^, 70^▼^, 47^▼^	WH	GC117
							Herpes zoster	L	F/D	D	Powderize then paint			
43.	Plumbaginaceae	*Plumbago zeylanica* L.	Amera	H	4	0.05	Hemorrhoid	L	F	D	Rub and tie	31^▼^, 38^▼^, 62^▲^, 79^▼^, 53^▼^, 40^▼^, 9^▼^, 63^▲^, 29^▲^, 70^▼^, 71^▼^59^▼^, 81^▲^	W	GC128
							Snake bite	R	F/D	D	Take in the pocket when moving			
							Wound	St,L	D	D	Burn and paint together with butter			
44.	Podocarpaceae	*Podocarpus falcatus* L.	Zigiba	T	1	0.01	Asthma	Sb	D	O	Powderize, mix water, and put it 7 days in the ground then drink	50^▼^, 40^▼^, 65^▼^, 70^▼^, 81^▼^	H	GC212
45.	Polygalaceae	*Polygala sphenoptera* Fresen.	Kibie zelzil	H	3	0.04	Snake poison, malaria	R	F/D	O	Peel, chew, and absorb the juice	–	W	GC208
							Stomachache	R	F/D	O	Chew and absorb the juice			
46.	Polygonaceae	*Rumex abyssinicus* Jacq.	Mekmeko	H	1	0.01	Expel delayed embryo in the uterus	R	D	O	Powderize the concoction and add water then drink	31^▼^, 38^▼^, 37^▼^, 63^▼^, 68^▲^, 50^▼^, 77^▲^, 46^▼^, 70^▼^, 78^▼^, 47^▼^	W	GC076
							Intestinal parasite	R	D	O	Powderize the concoction and add honey then drink. Drink Tella a few minutes later			
		*Rumex nepalensis* Spreng.	Tult	H	13	0.16	Over blood flow after birth	L	F	–	Cut and drop on ground	31^▼^, 42^▲^, 59^▼^, 63^▲^, 68^▲^, 50^▼^, 54^▲^, 46^▲^, 40^▼^, 65^▼^, 45^▲^, 78^▼^, 73^▼^	W	GC029
							Dandruff	R	F	D	Crush then wash with it			
							Stomachache	R	F	O	Crush and smash in, water then drink			
							Stomachache	R	F/D	O	Powderize the concoction and eat with butter			
							Bleeding and wound	L	F	D	Crush the concoction then tie/rub and tie alone			
		*Rumex nervosus* Vahl	Enbuacho	S	8	0.1	Warts	L	F/D	D	Crush the concoction, heat and put on the spot	31^▼^, 38^▼^, 50^▼^, 37^▼^, 63^▲^, 46^▼^, 78^▼^, 84^▼^	W	GC177
							Wound	L	F/D	D	Crush then tie			
							Fire burn	R	D	D	Powderize then paint			
47.	Portulacaceae	*Portulaca oleracea* L.	Antra	H	1	0.01	Hemorrhoid	L	F	D	Crush the concoction, mix with salt, and smash in water then tie on the spot	79^▼^, 32^▼^, 40^▼^	W	GC193
48.	Pteridiaceae	*Pteris dentata* Forsskal	Jero asfit	H	2	0.02	Eczema	L	F/D	D	Burn and paint the powder	–	W	
49.	Ranunculaceae	*Clematis simensis* Fresen.	Azo hareg	Cl	1	0.01	Cutaneous leishmaniasis	R	D	D	Powderize the concoction then paint together with honey	31^▼^, 63^▼^, 50^▼^, 78^▼^, 46^▲^, 40^▼^, 83^▼^	W	GC043
		*Ranunculus stagnalis* Hochst. Ex A. Rich.	Gudign	H	4	0.05	Eczema	L	F/D	D	Crush the concoction then paint with butter	–	W	GC182
							Wound	L	F	D	Mix ash with butter then paint			
		*Thalictrum rhynchocarpum* Dill. & A. Rich.	Sire-bizu	H	3	0.04	Stomachache	R	F	O	Crush then smash in water, filtrate and drink	63^▼^, 50^▼^, 40^▼^, 65^▼^	W	GC078
50.	Rhamnaceae	*Rhamnus prinoides* L’Her.	Gesho	S	18	0.21	Tonsillitis	L	F	O	Squeeze and drink	31^▲^, 38^▲^, 50^▲^, 57^▼^, 34^▼^, 37^▲^, 59^▼^, 63^▲^, 68^▲^, 65^▲^, 77^▲^, 29^▲^, 45^▼^, 47^▲^, 84^▲^	H	GC094
		*Ziziphus spina-christi* (L.) Desf.	Kunkura/orsamisa	S	8	0.1	Anthrax	L	F/D	O	Crush the concoction and add honey then eat	38^▲^, 79^▼^, 50^▼^, 51^▼^, 63^▲^, 77^▲^, 32^▼^, 75^▼^, 84^▲^	W	GC163
							Dandruff	L	F/D	D	Crush and paint			
51.	Rosaceae	*Hagenia abyssinica* (Bruce) J.F.Gmelin	Koso	T	3	0.04	Intestinal parasite	Ft	F/D	O	Crush then eat with honey	31^▲^, 54^▲^, 37^▲^, 50^▲^, 42^▲^, 59^▲^, 29^▲^, 65^▲^, 55^▲^, 70^▲^, 47^▲^, 82^▲^	WH	GC183
52.	Rubiaceae	*Coffea arabica* L.	Bunna	S	1	0.01	Asthma	Sd	D	O	Decoct and filtrate then drink	53^▼^, 40, ^▼^, 37^▲^, 63^▼^, 68^▼^, 67^▼^, 65^▼^, 80^▼^, 56^▼^, 35^▲^, 38^▼^	H	GC161
		*Galium simense* Fresen.	Ashekt	H	1	0.01	Tinea versicolor	L	F	D	Squeeze the concoction and paint until recovery	76^▲^	W	GC195
		*Rubia cordifolia* L.	Mencherer	Cl	6	0.06	Broken bonee	L	F	D	Crush then tie with butter	31^▼^, 62^▼^, 48^▲^, 50^▲^, 63^▲^, 46^▲^, 40^▼^, 71^▼^	W	GC110
							Cough, TB, lung cancer	R	D	O	Crush and smash in water in 3 days then drink			
							Cough	L	F/D	O	Powderize the concoction then drink with honey			
53.	Rutacea	*Citrus aurantifolia* (Christm.) Swingle	Lomy	S	5	0.06	Acne	Ft	F	D	Squeeze and paint the juice	49^▲^, 37^▼^, 3^▼^, 60^▼^, 36^▼^, 63^▼^, 68^▲^, 29^▲^, 46^▲^, 65^▼^, 45^▼^, 55^▼^, 58^▼^, 69^▲^, 35^▼^, 74^▼^, 70^▼^, 73^▲^, 83^▲^	H	GC169
							Stomach problem	Ft	F	O	Squeeze and drink			
							Tinea versicolor	L	F	D	Squeeze the concoction then paint			
		*Citrus medica* L.	Tirngo	T	1	0.01	Appetite	Ft	D	O	Powderize the concoction then drink the decoction	40^▼^, 60^▼^, 69^▲^	H	GC185
		*Clausena anisata* (Willd.) Benth.	Linbich	S	2	0.02	Evil spirit	R	D	DNO	Crush and fumigate	1^▼^, 48^▼^, 59^▼^, 76^▼^, 63^▼^, 46^▼^, 47^▲^, 81^▲^	W	GC235
		*Ruta chalepensis* L.	Tenadam	H	14	0.15	Stomachache	Ft, L	F	O	Chew and absorb the juice	31^▼^, 38^▲^, 54^▲^, 37^▲^, 42^▲^, 59^▲^, 63^▲^, 68^▲^, 50^▲^, 40^▼^, 77^▼^, 29^▲^, 46^▲^, 45^▲^, 70^▼^, 65^▼^, 78^▲^, 47^▲^, 82^▲^, 83^▲^, 84^▼^	H	GC236
							Evil eye	Ft,L,St	F/D	O	Chew and absorb the juice			
54.	Santalaceae	*Osyris quadripartita* Salzm. ex Decne	Keret	S	2	0.02	Rabies	R	F	O	Crush the concoction then drink with milk	31^▼^, 68^▼^, 79^▲^, 50^▼^, 78^▼^, 40^▼^, 46^▼^, 65^▼^	W	GC237
		*Thesium kilimandscharicum* Engl.	–	H	1	0.01	Dactylitis	All	F/D	D	Burn and paint the ash	–	W	GC218
55.	Sapindaceae	*Dodonaea angustifolia* L.f.	Kitkita	S	3	0.04	Trachoma	R	D	Op	Paint the concoction ash	31^▲^, 38^▲^, 54^▼^, 37^▼^, 50^▲^42, 63^▲^, 68^▼^, 46^▲^, 78^▲^, 40^▼^, 45^▲^, 55^▼^, 32^▼^, 65^▼^, 70^▼^, 73^▼^, 81^▼^	W	GC036
							Wound	L	F/D	D	Paint the ash			
							Eczema	L	D	D	Powderize then paint together with butter and expose to sunlight			
56.	Scrophulariaceae	*Verbascum sinaticum* Benth.	Kutitina	H	4	0.05	Stomachache	R	F	O	Peel, chew, and absorb the juice	31^▼^, 38^▲^, 79^▼^, 59^▲^, 63^▲^, 50^▼^, 78^▼^, 46^▼^, 65^▼^, 45^▲^, 70^▼^, 73^▼^	W	GC074
							Febrile illness	R	F	O	Crush, smash in water, and drink			
	Solanaceae	*Capsicum annuum* L.	Karia/keto	H	1	0.01	Malaria	Ft	F	O	Chop the concoction then eat	72^▼^, 37^▼^42, 63^▲^, 29^▲^, 46^▼^, 45^▼^	H	GC026
		*Datura stramonium* L.	Banje	H	14	0.17	Dandruff	L	F/D	D	Crush the concoction then paint/crush and paint alone	31^▲^, 38^▲^, 79^▼^, 37^▲^, 50^▼^, 59^▲^, 76^▼^, 63^▲^, 61^▼^, 68^▲^, 40^▲^, 77^▼^, 29^▼^, 46^▼^, 45^▲^, 70^▼^, 65^▼^, 78^▲^, 73^▼^, 47^▼^, 83^▲^, 84^▲^	WH	GC124
		*Discopodium penninervium* Hochst.	Segelet	S	2	0.02	Anthrax	L	F	O	Rub and squeeze then drink	29^▲^, 65^▼^	WH	GC188
							Stomachache	L	F	O	Squeeze and drink with water			
		*Lycopersicon esculentum* Mill.	Timatim	H	1	0.01	Malaria	L	F	O	Squeeze then drink	38^▼^, 78^▼^	WH	GC207
							Excessive bleeding after birth	L	F	Vl	Rub and insert in a cloth			
		*Nicotiana tabacum* L.	Tinbaho	S	1	0.01	Evil spirit	L	F	N	Rub and insert in the nose	31^▼^, 38^▼^, 50^▼^, 48^▼^, 63^▼^, 68^▼^, 46^▼^, 40^▼^, 45^▼^, 70^▼^, 65^▼^, 83^▲^	WH	GC080
		*Solanum incanum* L.	Edi	S	18	0.21	Wound	L	F/D	D	Crush then tie	31^▼^, 38^▲^, 34^▼^, 50^▼^, 53^▼^, 2^▼^, 76^▼^, 63^▲^, 68^▲^, 40^▲^, 77^▲^, 48^▲^, 29^▲^, 46^▲^, 45^▼^, 55^▼^, 66^▼^, 70^▼^, 73^▲^, 47^▲^, 81^▲^, 83^▼^	W	GC059
							Bleeding	L	F	N	Rub and sniff			
							Bloating	L	F	O	Chop and make Wote then eat with Enjera			
							Swelling	R	F	D	Crush and tie			
							Headache	R	F/D	DNO	Fumigate			
							Scorpion poison	R	F/D	O	Chew and absorb the juice			
							Stomachache	R	F	O	Peel, chew, and absorb the juice			
		*Solanum marginatum* L.f.	Geber enboy	S	2	0.02	Scorpion poison	R	F	O	Chew and absorb the juice	38^▼^, 50^▼^, 59^▼^, 46^▼^, 65^▼^, 73^▼^, 81^▲^	W	GC095
		*Solanum nigrum* L.	Awut	H	5	0.06	Dactylitis	L	F	D	Rub and tie	33^▲^, 49^▼^, 38^▼^, 63^▲^, 67^▼^, 66^▲^, 69^▲^, 71^▼^, 75^▲^, 82^▲^	W	GC140
							Wound	L	F/D	D	Crush and tie			
							Herpes zoster	L	F/D	D	Crush the concoction then paint			
							Bleeding	L	F	D	Squeeze and paint			
							Liver problem	L	F	O	Crush and make Wote with butter then eat with Enjera			
		*Withania somnifera* (L.) Dunal in DC.	Giziewa/ed ebudha	S	11	0.13	Evil spirit	L	F/D	DNO	Fumigate	31^▲^, 38^▲^, 79^▼^, 50^▲^, 78^▲^, 3^▼^, 48^▼^, 37^▲^, 76^▲^, 63^▲^, 68^▲^, 77^▲^, 29^▲^, 46^▼^, 45^▲^, 32^▲^, 70^▼^, 65^▼^, 73^▲^, 82^▲^, 83^▲^, 84^▼^	WH	GC048
							General ailments and epidemic	R and L	F/D	DNO	Rub the leaf then paint/burn the root and fumigate			
							Swelling	R	F/D	D	Crush and mix honey then tie/burn and add honey then paint			
57.	Tiliaceae	*Grewia ferruginea* Hochst. ex A. Rich.	Lenkuata	S	2	0.02	Asthma and stomachache	R,B	F/D	O	Powder, the concoction then drink with honey and butter	31^▼^, 54^▼^, 40^▼^, 42^▼^, 63^▼^, 77^▼^46^▼^, 32^▼^, 47^▼^, 83^▲^	W	GC123
		*Grewia kakothamnos* K.schum.	Tieka	S	2	0.02	Swelling	L	F	D	Chew and paint	–	W	GC198
58.	Ulmaceae	*Celtis africana* Burm.f	–	S	1	0.01	Dactylitis	L	F/D	D	Crush then tie	65^▼^	W	GC238
59.	Urticaceae	*Urtica simensis* Steudel	Sama	H	3	0.04	Warts	L	F	D	Squeeze then cream and rub on skin	50^▼^, 46^▼^	W	GC223
60.	Verbenaceae	*Verbena officinalis* L.	Atuch	H	1	0.01	Stomachache	R	F/D	O	Chew and swallow the juice	31^▲^, 38^▲^, 50^▼^, 59^▲^, 78^▼^, 63^▼^, 29^▲^, 70^▼^, 81^▼^	WH	GC069
61.	Vitaceae	*Cayratia gracilis* (Guill.&Perr.) Suesseng	Aserkush	H	2	0.02	Herpes zoster	L	F	D	Squeeze the concoction then paint	63^▼^	W	GC222
		*Cyphostemma cyphopetalum* (Fresen.)Desc. Ex Wild & Drumm.	Abawoldu	H	1	0.01	Eczema	L	F	D	Squeeze the concoction then paint	64^▼^, 70^▼^	W	GC234
		*Rhoicissus tridentata* (L.f)Wild & Drumm.	Este haregawin	Cl	2	0.02	Brain weakness	R	F/D	O	Powderize the concoction and mix honey then drink the decoction	38^▼^, 62^▼^	W	GC230

Habit: *H* herb, *T* tree, *S* shrub, *Cl* climber. Pu (plant parts used): *L* leaf, *R* root, *B* bark, *Lx* latex, *Ft* fruit, *Fr* flower, *Sd* seed, *Sb* stem bark, *Rb* root bark. Cp (condition of preparation): *F* fresh, *D* dry, *F/D* fresh or dry. *RA* route of administration: *D* dermal, *O* oral, *N* nasal; *DNO* dermal, nasal, and oral; *Er* ear; *Op* optical; *Vl* vaginal. Comparison with similar studies: ^▲^ similar uses, ^▼^ dissimilar uses, − no similar documentation found in the reviews. Ht (habitat): *W* wild, *H* home garden, *WH* wild and home garden

**Table 11:** Comparative analysis of the uses of medicinal plant species recorded in this study with other similar studies undertaken in different places

Sample no	Study area and references	Total no. of species reported	No. of similar uses	No. of dissimilar uses	Percentages of new uses reported in the present study
1.	Loma and Gena bosa Districts, southern Ethiopia [68]	158	16	24	15.19
2.	Seharti Samre District, Southern Tigray, Ethiopia [38]	87	25	27	31.03
3.	Guji Oromo Tribes in Abaya District, Borana, Oromia, Ethiopia [10]	43	8	11	25.58
4.	Harla and Dengego valleys, eastern Ethiopia [79]	83	2	14	16.87
5.	Erer Valley of Babile Wereda, Eastern Ethiopia [32]	51	1	11	21.57
6.	Gondar Zuria District, Northwestern Ethiopia [37]	42	12	16	38.10
7.	Libo Kemkem District, Northwest Ethiopia [63]	149	34	35	23.49
8.	In and around Fiche District, Central Ethiopia [50]	106	11	43	40.57
9.	Debre Libanos Wereda, Central Ethiopia [78]	77	9	32	41.56
10.	Bench ethnic group of Ethiopia [61]	35	1	15	42.86
11.	Ada’a District, East Shewa Zone of Oromia Regional State, Ethiopia [46]	112	16	39	34.82
12.	Maale and Ari ethnic communities in southern Ethiopia [29]	128	15	18	14.06
13.	Mana Angetu District, Southeastern Ethiopia [40]	203	4	41	20.20
14.	Ankober District, North Shewa Zone, Amhara Region, Ethiopia [65]	135	8	41	30.40
15.	Wayu Tuka District, Oromia Regional State, West Ethiopia [47]	103	8	16	15.53
16.	Wonago Woreda, SNNPR, Ethiopia [42]	58	9	13	22.41
17.	Amaro Woreda, Ethiopia [45]	56	13	16	28.57
18.	Hawassa city, Southern Ethiopia [70]	80	7	39	48.75
19	Kilte Awulaelo District, Tigray Region of Ethiopia [73]	106	10	22	20.75
20.	Zegie Peninsula, Northwestern Ethiopia [59]	67	12	16	23.88
21.	Ethiopia [75]	35	2	5	14.29
22.	Sekoru Woreda, Jimma Zone, Southwestern Ethiopia [76]	27	3	15	55.56
23.	Asgede Tsimbila district, Northwestern Tigray, northern Ethiopia [77]	68	8	23	33.82
24.	Gindeberet District,Western Ethiopia [55]	26	6	11	42.31
25.	South africa [62]	46	2	2	4.35
26.	Abeokuta areas of Ogun State, Nigeria [36]	58		6	10.35
27.	Igede people of Nigeria [1]	90	–	3	3.33
28.	Ogun state of Nigeria [60]	36	–	2	5.56
29.	Gomari Airport Ward, Jere local government area, Borno State, Nigeria [30]	22	–	3	13.64
30.	Southwestern Nigeria [72]	143	1	7	4.9
31.	Nakapiripirit, Pallisa, Kanungu, and Mukono in Uganda [64]	262	–	1	0.38
32.	Uganda [53]	103	–	12	11.65
33.	Maasai of Southern Kaijiado District, Kenya [2]	41	1	1	2.44
34.	Kikuyu, Central Kenya [3]	58	–	3	5.17
35.	Sabaots of mt.elgon Kenya [48]	107	3	15	14.02
36.	Eastern Desert of Egyp Wadi El-Gemal National Park [51]	70	–	3	4.29
37.	Beregadougou and Fabedougou, Cascades Region, Burkina Faso [33]	95	–	4	4.21
38.	Bhopal district, India [49]	79	–	7	8.86
39.	Coochbehar district, West Bengal, India [9]	46	–	3	6.52
40.	Keelakodankulam village, South India [67]	70	3	4	5.71
41.	Irula Tribe of Hasanur Hills, Erode District, India [71]	70	–	6	8.57
42.	Nandi, Kenya [57]	44	2	3	6.82
43.	Conakry and Dubreka, Guinean [34]	67	–	3	4.48
44.	Kadipur village of Chuadanga District, Bangladesh [58]	33	1	3	9.09
45.	Sylhet Division, Bangladesh [69]	107	3	2	1.87
46.	Province of Camagüey, Cuba [74]	123	–	2	1.63
47.	Izmir Province, Turkey [56]	33	–	1	3.03
48.	District Mirpur, AJK, Pakistan [66]	29	2	3	10.35
49.	Tabarkins, Northern Italy [80]	53	1	8	15.09
50.	Itapoa, Southern Brazil [35]	109	3	2	1.83
51.	Dek Island in Ethiopia [81]	60	15	10	16.7
52.	Zay people in Ethiopia [82]	33	7	6	18.18
53.	Dheeraa town, Arsi Zone, Ethiopia [83]	83	13	16	19.28
54.	Around Alamata, Southern Tigray, Northern Ethiopia [84]	25	9	7	28
55.	Mecha Wereda West Gojjam Zone of Ethiopia [31]	102	16	30	29.41
		Total average mean percentages of new uses			17.85

## References

[1] Igoli JO, Ogaji OG, Tor-Anyiin TA, Igoli NP (2005). Traditional medicine practice amongst the Igede people of Nigeria. Afr J Trad CAM.

[2] Kiringe JW (2006). A survey of traditional health remedies used by the Maasai of Southern Kaijiado District, Kenya. Ethnobot Res Appl.

[3] Njoroge GN, Bussmann RW (2006). Diversity and utilization of antimalarial ethnophytotherapeutic remedies among the Kikuyus (Central Kenya). J Ethnobiol Ethnomed.

[4] Kibebew F, Zewdu M, Demissie A (2001). The status and availability of oral and written knowledge on traditional healthcare in Ethiopia. Conservation and sustainable use of medicinal plants in Ethiopia. Proceedings of National Workshop on Biodiversity Conservation and Sustainable Use of Medicinal Plants in Ethiopia. 28 April to 01 May 1998.

[5] WHO (2002). Traditional medicine: growing needs and potentials.

[6] Abebe D (1998). Traditional medicine in Ethiopia: the attempts being made to promote it for effective and better utilization. SINET: Ethiop J Sci.

[7] Abebe D, Ayehu A (1993). Medicinal plants and enigmatic health practices of Northern Ethiopia.

[8] WHO (2000). General guidelines for methodologies on research and evaluation of traditional medicine.

[9] Datta T, Patra AK, Dastidar SG (2014). Medicinal plants used by the tribal population of Coochbehar district, West Bengal, India-an ethnobotanical survey. Asian Pac J Trop Biomed.

[10] Bekele G, Reddy PR (2015). Ethnobotanical study of medicinal plants used to treat human ailments by Guji Oromo tribes in Abaya District, Borana, Oromia, Ethiopia. Universal J Plant Sci.

[11] GDAO (2007). Climatic and geographical recorded data about Gubalafto District. Gubalafto District administration office.

[12] CSA (2007). The 2007 Population and Housing Census for Ethiopia, statistical report results at country level.

[13] GDHHCO (2007). Human diseases data. Gubalafto District human health care office.

[14] Martin GJ (1995). Ethnobotany: a method manual.

[15] Cotton CM (1996). Ethnobotany: principles and applications.

[16] Alexiades M, Alexiades M, Sheldon JW (1996). Collecting ethnobotanical data: an introduction to basic concepts and techniques. Selected guideline for ethnobotanical research: a field manual.

[17] Edwards S, Demissew S, Hedberg I, editors. Flora of Ethiopia and Eritrea. Hydrocharitaceae to Arecaceae volume 6. Ethiopia: Department of Systematic Botany, Uppsala University, Uppsala and The National Herbarium, Addis Ababa University, Addis Ababa; 1997.

[18] Edwards S, Tadesse M, Demissew S, Hedberg I, editors. Flora of Ethiopia and Eritrea. Magnoliaceae to Flacourtiaceae volume 2 part 1. Ethiopia: Department of Systematic Botany, Uppsala University, Uppsala and The National Herbarium, Addis Ababa University, Addis Ababa; 2000.

[19] Edwards S, Tadesse M, Hedberg I, editors. Flora of Ethiopia and Eritrea. Canellaceae to Euphorbiaceae part 2 volume 2. Ethiopia: Department of Systematic Botany, Uppsala University, Uppsala and The National Herbarium, Addis Ababa University, Addis Ababa; 1995.

[20] Hedberg I, Edwards S, editors. Flora of Ethiopia and Eritrea. Poaceae (Gramineae) volume 7. Ethiopia: Department of Systematic Botany, Uppsala University, Uppsala and The National Herbarium, Addis Ababa University, Addis Ababa; 1995.

[21] Hedberg I, Edwards S, editors. Flora of Ethiopia and Eritrea. Pittosporaceaeto Araliaceae volume 3. Ethiopia: Department of Systematic Botany, Uppsala University, Uppsala and The National Herbarium, Addis Ababa University, Addis Ababa; 1989.

[22] Hedberg I, Edwards S, Nemomissa S, editors. Flora of Ethiopia and Eritrea. Apiaceae to Dipsacaceae volume 4 part 1. Ethiopia: Department of Systematic Botany, Uppsala University, Uppsala and the National Herbarium, Addis Ababa University, Addis Ababa; 2003.

[23] Hedberg I, Friis I, Edwards S, editors. Flora of Ethiopia and Eritrea. Asteraceae Part 2 Volume 4. Ethiopia: Department of Systematic Botany, Uppsala University, Uppsala and the National Herbarium, Addis Ababa University, Addis Ababa; 2004.

[24] Hedberg I, Friis I, Person E, editors. Flora of Ethiopia and Eritrea. Lycopodiaceae to Pinaceae. Volume 1. Ethiopia: Department of Systematic Botany, Uppsala University, Uppsala and The National Herbarium, Addis Ababa University, Addis Ababa; 2009.

[25] Hedberg I, Kelbessa E, Edwards S, Demissew S, Persson E, editors. Flora of Ethiopia and Eritrea. Plantaginaceae volume 5. Ethiopia: Department of Systematic Botany, Uppsala University, Uppsala and The National Herbarium, Addis Ababa University, Addis Ababa; 2006.

[26] Heinrich M, Ankli A, Frei B, Weimann C, Sticher O (1998). Medicinal plants in Mexico: healers’ consensus and cultural importance. Soc Sci Med.

[27] Phillips O, Gentry AH, Reynel C, Wilkin P, Galvez-Durand BC (1994). Quantitative ethnobotany and Amazonian conservation. Conserv Biol.

[28] Friedman J, Yaniva Z, Dafnib A, Palewitch D (1986). A preliminary classification of the healing potential of medicinal plants, based on a rational analysis of an ethnopharmacological field survey among Bedouins in the Negev Desert, Israel. J Ethnopharmacol.

[29] Kidane B, Van Ande T, Josephus L, van der Maesen G, Asfaw Z (2014). Use and management of traditional medicinal plants by Maale and Ari ethnic communities in southern Ethiopia. J Ethnobiol Ethnomed.

[30] Sodipo OA, Wannang NN (2015). Ethnopharmacological survey of plants used by trado-medical practitioners (TMPs) in the treatment of typhoid fever in Gomari Airport Ward, Jere local government area, Borno State, Nigeria. Am J Ethnomed.

[31] Gebeyehu G, Asfaw Z, Enyew A, Raja N (2014). Ethnobotanical study of traditional medicinal plants and their conservation status in Mecha Wereda West Gojjam Zone of Ethiopia. Int J Pharm H Care Res.

[32] Belayneh A, Asfaw Z, Demissew S, Bussa NF (2012). Medicinal plants potential and use by pastoral and agro-pastoral communities in Erer Valley of Babile Wereda, Eastern Ethiopia. J Ethnobiol Ethnomed.

[33] Sourabie TS, Kinda D, Yaro B, Nikiema JB (2013). Ethnobotanical survey of medicinal plants used by the traditional medical healers in the villages of Beregadougou and Fabedougou (Cascades Region, Burkina Faso). IOSR J Pharm.

[34] Balde AM, Traore MS, Diallo MST, Balde ES, Huang Y, Liu Z, Oulare K, Barry MS, Balde MA, Camara A, Berghe DV, Vlietinck A, Pieters L (2015). Ethnobotanical survey, antimicrobial and anticomplementary activities of Guinean medicinal plants traditionally used in the treatment of inflammatory diseases in Conakry and Dubreka. J Plant Sci.

[35] Meretika AHC, Peroni N, Hanazaki EN (2010). Local knowledge of medicinal plants in three artisanal fishing communities (Itapoa, Southern Brazil), according to gender, age, and urbanization. Acta Bot Bras.

[36] Erinoso SM, Aworinde DO (2012). Ethnobotanical survey of some medicinal plants used in traditional health care in Abeokuta areas of Ogun State, Nigeria. Afr J Pharm Pharmacol.

[37] Birhanu Z (2013). Traditional use of medicinal plants by the ethnic groups of Gondar Zuria District, north-western Ethiopia. J Nat Remed.

[38] Araya S, Abera B, Giday M (2015). Study of plants traditionally used in public and animal health management in Seharti Samre District, southern Tigray, Ethiopia. J Ethnobiol Ethnomed.

[39] Flatie T, Gedif T, Asres K, Gebre-Mariam T (2009). Ethnomedical survey of Berta ethnic group Assosa Zone, Benishangul-Gumuz regional state, mid-west Ethiopia. Ethnobiol Ethnomed.

[40] Lulekal E, Kelbessa E, Bekele T, Yineger H (2008). An ethnobotanical study of medicinal plants in Mana Angetu District, southeastern Ethiopia. J Ethnobiol Ethnomed.

[41] Awas T, Demissew S. Ethnobotanical study of medicinal plants in Kafficho people, southwestern Ethiopia. In: Proceedings of the 16th International Conference of Ethiopian Studies edited by Ege V, Aspen H, Teferra B, Bekele S. Trondheim; 2009.

[42] Mesfin F, Demissew S, Teklehaymanot T (2009). An ethnobotanical study of medicinal plants in Wonago Woreda, SNNPR, Ethiopia. J Ethnobiol Ethnomed.

[43] Yalew A, Debebe Y, Ashok PK, Zewdneh T, Assefa A (2012). Traditional medicinal plants used by people in Libo-Kemkem District, South Gondar, Ethiopia. Asian J Agric Sci.

[44] Abera B (2014). Medicinal plants used in traditional medicine by Oromo people, Ghimbi District, Southwest Ethiopia. Ethnobiol Ethnomed.

[45] Mesfin F, Seta T, Assefa A (2014). An ethnobotanical study of medicinal plants in Amaro Woreda, Ethiopia. Ethnobotany Res Appl.

[46] Kefalew A, Asfaw Z, Kelbessa E (2015). Ethnobotany of medicinal plants in Ada’a District, East Shewa Zone of Oromia Regional State, Ethiopia. J Ethnobiol Ethnomed.

[47] Megersa M, Asfaw Z, Kelbessa E, Beyene A, Woldeab B (2013). An ethnobotanical study of medicinal plants in Wayu Tuka District, East Welega Zone of Oromia Regional State, West Ethiopia. J Ethnobiol Ethnomed.

[48] Okello SV, Nyunja RO, Netondo GW, Onyango JC (2010). Ethnobotanical study of medicinal plants used by Sabaots of Mt. Elgon Kenya. Afr J Trad CAM.

[49] Agarwal K, Varma R (2012). Some ethnomedicinal plants of Bhopal district used for treating stone diseases. Int J Pharm Life Sci.

[50] Enyew A, Asfaw Z, Kelbessa E, Nagappan R (2014). Ethnobotanical study of traditional medicinal plants in and around Fiche District, Central Ethiopia. Curr Res J Biol Sci.

[51] Mahmoud T, Gairola S (2013). Traditional knowledge and use of medicinal plants in the Eastern Desert of Egypt: a case study from Wadi El-Gemal National Park. J Med Plants Stud.

[52] Hailemariam TB, Demissew SW, Asfaw ZW (2009). An ethnobotanical study of medicinal plants used by local people in the lowlands of Konta Special Woreda, southern nations, nationalities and peoples regional state, Ethiopia. Ethnobiol Ethnomed.

[53] Lamorde M, Tabuti JRS, Obua C, Kukunda-Byobona C, Lanyero H, Byakika-Kibwika P, Bbosa GS, Lubega A, Ogwal-Okeng J, Ryan M, Waako PJ, Merry C. Medicinal plants used by traditional medicine practitioners for the treatment of HIV/AIDS and related conditions in Uganda. J Ethnopharmacol. doi:10.1016/j.jep.2010. 10.1016/j.jep.2010.04.004 20451595

[54] Yineger H, Kelbessa E, Bekele T, Lulekal E (2008). Plants used in the traditional management of human ailments at Bale Mountain National Park, southeastern Ethiopia. J Med Plant Res.

[55] Zerabruk S, Yirga G (2012). Traditional knowledge of medicinal plants in Gindeberet District, western Ethiopia. S Afr J Bot.

[56] Dogan Y, Ugulu I (2013). Medicinal plants used for gastrointestinal disorders in some districts of Izmir Province, Turkey. Ethno Med.

[57] Pascaline J, Charles M, George O, Lukhoba C (2011). An inventory of medicinal plants that the people of Nandi use to treat malaria. J Anim Plant Sci.

[58] Chowdhury HKASM, Shahriar H, Rahman S, Uddin P, Al-Amin RM, Bhuiyan TA, Afrin S, Chowdhury S, Rahman M, Azad AK, Rahmatullah M (2015). Home remedies of rural folks: a study in Kadipur village of Chuadanga District, Bangladesh. World J Pharmacy Pharm Sci.

[59] Teklehaymanot T, Giday M (2007). Ethnobotanical study of medicinal plants used by people in Zegie Peninsula, northwestern Ethiopia. J Ethnobiol Ethnomed.

[60] Ogbole OO, Ajaiyeoba EO (2010). Traditional management of tuberculosis in the Ogun state of Nigeria: the practice and ethnobotanical survey. Afr J Trad CAM.

[61] Giday M, Asfaw Z, Woldu Z, Teklehaymanot T (2009). Medicinal plant knowledge of the bench ethnic group of Ethiopia: an ethnobotanical investigation. Ethnobiol Ethnomed.

[62] Abdillahi HS, Van Staden J (2012). South African plants and male reproductive healthcare: conception and contraception. J Ethnopharmacol.

[63] Chekole G, Asfaw Z, Kelbessa E (2015). Ethnobotanical study of medicinal plants in the environs of Tara-gedam and Amba remnant forests of Libo Kemkem District, northwest Ethiopia. J Ethnobiol Ethnomed.

[64] John RS, Kukunda CB, Kaweesi D, Kasilo OMJ (2012). Herbal medicine use in the districts of Nakapiripirit, Pallisa, Kanungu, and Mukono in Uganda. J Ethnobiol Ethnomed.

[65] Lulekal E, Asfaw Z, Kelbessa E, Van Damme P (2013). Ethnomedicinal study of plants used for human ailments in Ankober District, North Shewa Zone, Amhara Region, Ethiopia. Ethnobiol Ethnomed.

[66] Mahmood A, Qureshi RA, Mahmood A, Sangi Y, Shaheen H, Ahmad I, Nawaz Z (2011). Ethnobotanical survey of common medicinal plants used by people of District Mirpur, AJK, Pakistan. J Med Plants Res.

[67] Mary DA, Franco FM, Babu V (2011). Assessing the contribution of local and traded biodiversity in community health care: a case study from Keelakodankulam village, South India. Ethnobotany Res Appl.

[68] Agize M, Demissew S, Asfaw Z (2013). Ethnobotany of medicinal plants in Loma and Gena bosa districts (woredas) of the Dawro zone, southern Ethiopia. Top Class J Herbal Med.

[69] Rahmatullah M, Khatun A, Morshed N, Neogi PK, SUA K, Hossan S, Maha MJ, Jahan R (2010). A randomized survey of medicinal plants used by folk medicinal healers of Sylhet Division, Bangladesh. Adv Nat Appl Sci.

[70] Regassa R (2013). Assessment of indigenous knowledge of medicinal plant practice and mode of service delivery in Hawassa city, southern Ethiopia. J Med Plant Res.

[71] Revathi P, Parimelazhagan T (2010). Traditional knowledge on medicinal plants used by the Irula tribe of Hasanur Hills, Erode District, Tamil Nadu, India. Ethnobotanical Leaflets.

[72] Soladoye MO, Adetayo MO, Chukwuma EC, Adetunji AN (2010). Ethnobotanical survey of plants used in the treatment of hemorrhoids in south-western Nigeria. Ann Biol Res.

[73] Teklay A, Abera B, Giday M (2013). An ethnobotanical study of medicinal plants used in Kilte Awulaelo District, Tigray region of Ethiopia. J Ethnobiol Ethnomed.

[74] Volpato G, Godinez D, Beyra A, Barreto A (2009). Uses of medicinal plants by Haitian immigrants and their descendants in the province of Camagüey, Cuba. J Ethnobiol Ethnomed.

[75] Yadav RH (2013). Medicinal plants in folk medicine system of Ethiopia. J Poisonous Med Plants Res.

[76] Yineger H, Yewhalaw D (2007). Traditional medicinal plant knowledge and use by local healers in Sekoru Woreda, Jimma Zone, southwestern Ethiopia. J Ethnobiol Ethnomed.

[77] Zenebe G, Zerihun M, Solomon Z (2012). An ethnobotanical study of medicinal plants in Asgede Tsimbila district, northwestern Tigray, northern Ethiopia. Ethnobotany Res Appl.

[78] Getaneh S, Girma Z (2014). An ethnobotanical study of medicinal plants in Debre Libanos Wereda, Central Ethiopia. African J Plant Sci.

[79] Belayneh A, Bussa NF (2014). Ethnomedicinal plants used to treat human ailments in the prehistoric place of Harla and Dengego valleys, eastern Ethiopia. J Ethnobiol Ethnomed.

[80] Maxia A, Lancioni MC, Balia AN, Alborghetti R, Pieroni A, Loi MC (2008). Medical ethnobotany of the Tabarkins, a Northern Italian (Ligurian) minority in south-western Sardinia. Genet Resour Crop Evol.

[81] Teklehymanot T (2009). Ethnobotanical study of knowledge and medicinal plants use by the people in Dek Island in Ethiopia. J Ethnophrmacology.

[82] Giday M, Asfaw Z, Elmqvist, Woldu Z (2003). An ethnobotanical study of medicinal plants used by the Zay people in Ethiopia. J Ethnophrmacology.

[83] Wondimu T, Asfaw Z, Kelbessa E (2007). Ethnobotanical study of medicinal plants around Dheeraa town Arsi Zone Ethiopia. J Ethnophrmacology.

[84] Yirga G (2010). Ethnobotanical study of medicinal plants in and around Alamata, Southern Tigray, Northern Ethiopia. Curr Res J Biol Sci.

